# Targeting fatty acid synthase in preclinical models of TNBC brain metastases synergizes with SN-38 and impairs invasion

**DOI:** 10.1038/s41523-024-00656-0

**Published:** 2024-06-10

**Authors:** Habib A. Serhan, Liwei Bao, Xu Cheng, Zhaoping Qin, Chia-Jen Liu, Jason A. Heth, Aaron M. Udager, Matthew B. Soellner, Sofia D. Merajver, Aki Morikawa, Nathan M. Merrill

**Affiliations:** 1 Department of Internal Medicine, University of Michigan, Ann Arbor, MI 48109 USA; 2 Department of Neurosurgery, University of Michigan, Ann Arbor, MI 48109 USA; 3 Department of Pathology, University of Michigan, Ann Arbor, MI 48109 USA; 4 Department of Chemistry, University of Michigan, Ann Arbor, MI 48109 USA

**Keywords:** Breast cancer, Breast cancer

## Abstract

Fatty acid synthesis (FAS) has been shown to play a key role in the survival of brain-metastatic (BM) breast cancer. We demonstrate that the fatty acid synthase inhibitor TVB-2640 synergizes with the topoisomerase inhibitor SN-38 in triple-negative breast cancer (TNBC) BM cell lines, upregulates FAS and downregulates cell cycle progression gene expression, and slows the motility of TNBC BM cell lines. The combination of SN-38 and TVB-2640 warrants further consideration as a potential therapeutic option in TNBC BMs.

## Introduction

Patients with triple-negative breast cancer (TNBC) and human epidermal growth factor receptor 2 (HER2) overexpressing breast cancer are at a higher risk for developing brain metastases (BMs) compared to patients with hormone receptor-positive, HER2-negative tumors^1–3^. BMs are challenging to treat in general and are associated with poor outcomes, especially for patients with TNBC BMs, who demonstrate the poorest overall survival due to a lack of effective molecular therapies, following progression on first-line therapy. The blood-brain barrier (BBB) modulates access to certain nutrients, such as fatty acids and amino acids to the brain microenvironment, and this imposes metabolic constraints for metastatic cancer cells, as they colonize the brain and undergo adaptations of signaling required for survival in the brain tissue environment^4,5^. Fatty acid synthesis (FAS) is a requirement for HER2+ breast cancer BM survival in the lipid-poor brain microenvironment^6^. Moreover, numerous studies have investigated the role of FAS enzymes in cancer, highlighting them as promising targets in breast, lung, and pancreatic cancers, among others^6–15^. To date, there are no fatty acid synthase (FASN) inhibitors approved for cancer treatment. However, the FASN inhibitor TVB-2640 is currently being examined in multiple phase II clinical trials, including a trial for patients with HER2+ breast cancer (NCT03179904), and has been shown to have a manageable toxicity profile in patients with and without brain tumors^16,17^.

To address the unmet need for effective therapeutic options for patients with TNBC BM, we explored TVB-2640 in this aggressive subtype of cancer. In particular, we hypothesized that modulation of FASN would offer an opportunity to exploit the vulnerability of cancer cells growing in a lipid-poor environment, when added to a topoisomerase I inhibitor, as potentially orthogonal mechanisms of action. In our study, we utilize the TNBC cell line MDA-MB-231, its brain-seeking subclone, MDA-MB-231-BR^18^, and two lab-generated cell lines, PDO-BC25 and PDO-BC25-2, developed from patient-derived organoids (PDOs) generated from TNBC BM resections at the University of Michigan. The PDOs were generated sequentially from a patient who had surgery as the initial treatment for BM and at the time of recurrence 6 months later (Supp. Table 1).

We performed drug-screening assays with three FASN inhibitors, TVB-2640, TVB-3166, and BI-99179, alone and in combination with the DNA topoisomerase I inhibitor SN-38, testing for synergy using the Chou-Talalay (CT) method^19,20^. To verify target engagement, we investigated the effects of these FASN inhibitors on gene expression using a panel of 768 metabolism genes. Finally, we performed invasion and migration assays in the presence and absence of TVB-2640 to determine the phenotypical impact of lipid synthesis modulation on cancer cell motility.

**Fig. 1:**
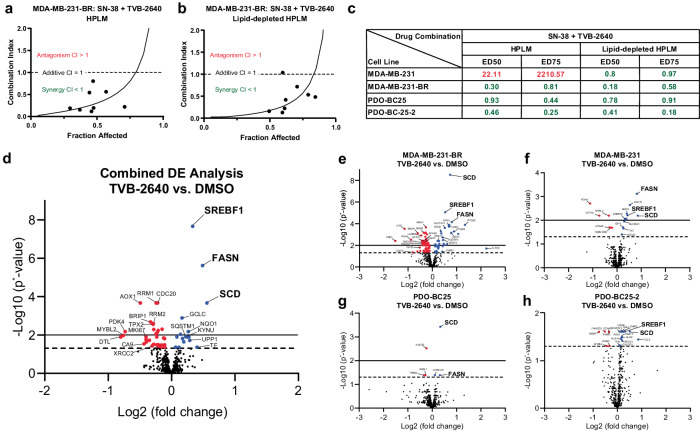
FASN inhibition with TVB-2640 synergizes with SN-38 and demonstrates evidence of target engagement in TNBC BM cell lines. Combination index plots were generated for the SN-38 + TVB-2640 combination in MDA-MB-231-BR in 3D culture using HPLM supplemented with (**a**) FBS or (**b**) lipid-depleted FBS. **c** Combination index values were determined at therapeutically relevant doses (ED50 and ED75) of SN-38 in combination with TVB-2640 in the four TNBC cell lines in 3D culture. CI < 1 indicates synergy, CI = 1 indicates additivity, and CI > 1 indicates antagonism. Volcano plots were generated showing DE analysis for a panel of metabolic genes using RNA collected from spheroids treated with 1 µM TVB-2640 vs. DMSO in HPLM supplemented with lipid-depleted FBS in 3D culture. **d** Combined DE analysis for all four cell lines was plotted in addition to individual plots for (**e**) MDA-MB-231-BR, (**f**) MDA-MB-231, (**g**) PDO-BC25, and (**h**) PDO-BC25-2. *N* = 4 biological replicates per cell line per treatment. Dashed line denotes *p*-adjusted = 0.05 and solid line denotes *p*-adjusted = 0.01 by the Benjamini–Hochberg method.

Sacituzumab govitecan (SG) is currently approved for patients with metastatic TNBC^21^. It is an antibody-drug conjugate that uses the topoisomerase I inhibitor SN-38 as its payload^22,23^. The drug has been shown to cross the BBB and is currently being examined in a clinical trial for patients with HER2-negative breast cancer BMs (NCT04647916). Moreover, a previous study showed a synergistic effect in a breast cancer cell line when combining the FASN inhibitor cerulenin with SN-38^9^. Therefore, we used the CT method to test the combination of SN-38 with FASN inhibition. Briefly, the CT method predicts additivity, relying on the dose-response curves of each drug individually, then compares the predicted additive effect to the actual effect of the drug combination and calculates combination index (CI) values for each combined dose to determine and quantify synergism, additivity, or antagonism^19,20^. The combinations were tested with cells cultured in base media supplemented with either fetal bovine serum (FBS) or lipid-depleted FBS, to isolate the effects of the FASN inhibitors while controlling lipid availability. Screening FASN inhibitors alone in vitro revealed low toxicity to the cell lines (Supp. Fig. 1). However, combining SN-38 with each FASN inhibitor revealed a synergistic effect in MDA-MB-231-BR, PDO-BC25, and PDO-BC25-2 (Fig. 1a–c and Supp. Table 2).

We then sought to explore how FASN inhibition impacts metabolism, specifically within the FAS pathway. To mimic the lipid-poor brain microenvironment, we supplemented the base medium with lipid-depleted FBS. We collected RNA from MDA-MB-231-BR cells treated with vehicle control or TVB-2640, then used NanoString rapid RNA-profiling to quantify RNA expression for a panel of 768 metabolic genes, and subsequently calculated differential expression between the conditions, under corrections for false discovery rate-adjusted *p*-values (p*). The analysis revealed a significant upregulation in stearyl-CoA desaturase (*SCD*) (p* < 0.01) in cells treated with TVB-2640 relative to DMSO (Supp. Fig. 2). *FASN* and sterol regulatory element binding factor 1 (*SREBF1*) were upregulated with FASN inhibition, but only significantly at the 10 μM dose of TVB-2640 (p* < 0.05 per gene). These initial experiments suggest that MDA-MB-231-BR may upregulate fatty acid synthesis genes to compensate for FASN inhibition when treated with TVB-2640.

Next, we cultured MDA-MB-231-BR, MDA-MB-231, PDO-BC25, and PDO-BC25-2 in the same lipid-poor media and treated the cells with DMSO or 1 μM TVB-2640. Differential expression analysis of all four cell lines together revealed significant upregulation in 16 genes involved in FAS, reactive oxygen species response, pentose phosphate pathway, nucleotide salvage, tryptophan-kynurenine metabolism, and autophagy. Significant downregulation was revealed in 31 genes involved in nucleotide synthesis, cell cycle progression, and the DNA damage response (Fig. 1d). Each cell line individually showed a significant upregulation in pro-FAS genes (Fig. 1e, f). Moreover, we performed the same analyses with TVB-3166 and BI-99179 and noted similar target engagement as TVB-2640, the FASN inhibitor under current evaluation in phase II clinical trials (Supp. Fig. 3). Lists of all significant differentially expressed genes may be found in Supplemental Table 3.

**Fig. 2:**
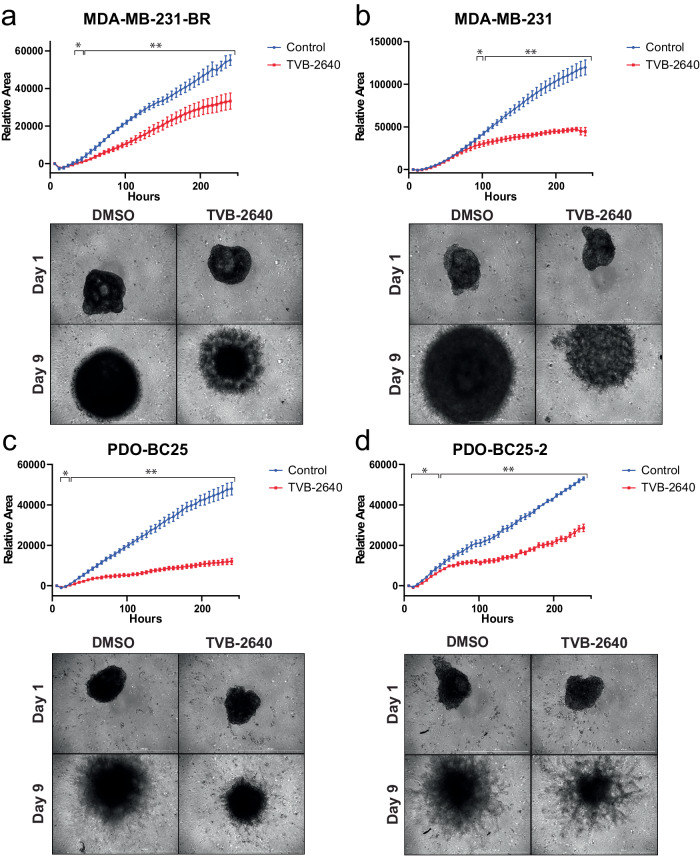
FASN inhibition with TVB-2640 impairs spheroid outgrowth in TNBC BM cell lines. Matrigel-embedded TNBC spheroids were treated with 1 μM TVB-2640 or DMSO and wells were imaged over 9 days to measure spheroid outgrowth in the TNBC cell lines: (**a**) MDA-MB-231-BR, (**b**) MDA-MB-231, (**c**) PDO-BC25, and (**d**) PDO-BC25-2. *N* = 6 replicates were seeded for each cell line and treatment condition. Error bars represent SEM. * denotes *p*-adjusted < 0.05 and ** denotes *p*-adjusted <0.01 by multiple unpaired *t*-tests. Scale bars indicate 1000 μm.

**Fig. 3:**
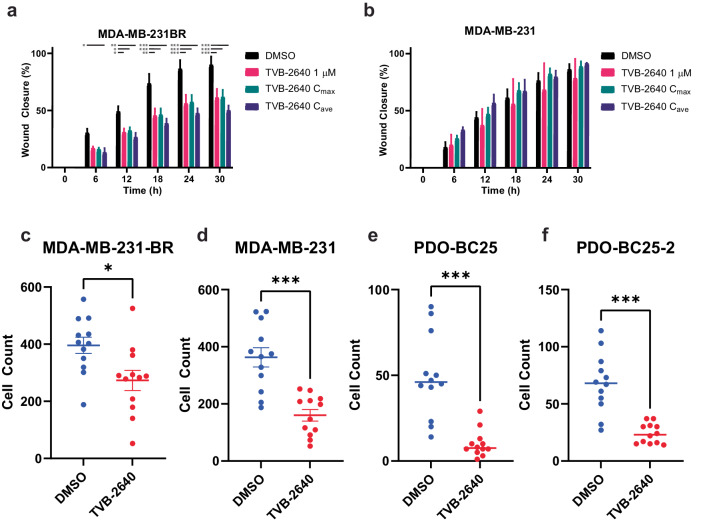
FASN inhibition with TVB-2640 slows cell migration and decreases invasion. Confluent cells were scratched and treated with 1 µM, *C*_ave_, or *C*_max_ concentrations of TVB-2640 vs. DMSO control. Imaging was performed every 6 h over 30 h to measure wound healing for (**a**) MDA-MB-231-BR and (**b**) MDA-MB-231. 6 images were captured per cell line and condition and *N* = 2–6 regions of interest were analyzed per cell line treatment, omitting wells where large masses of cells settled into the scratched area or images were out of focus. Error bars represent SEM. Additionally, cells were seeded in Matrigel-coated transwell inserts and treated with 1 µM TVB-2640 or DMSO. Invading cells were fixed, stained, and counted 24 h later and plotted for: (**c**) MDA-MB-231-BR; (**d**) MDA-MB-231; (**e**) PDO-BC25; and (**f**) PDO-BC25-2. *N* = 3 replicates per cell line per treatment where 4 regions of each replicate were imaged. Error bars denote SEM. * denotes *p* < 0.05, ** denotes *p* < 0.01, and *** denotes *p* < 0.001 by multiple unpaired two-tailed *t*-test.

Radial outgrowth is considered a robust surrogate for early steps of metastatic progression, and it is measurable in a distinct visual in vitro assay. To determine the phenotypic effects of TVB-2640 treatment on TNBC cell lines, we tested the outgrowth of Matrigel-embedded spheroids from each of the four cell lines treated with 1 μM TVB-2640 versus DMSO control. The outgrowth of the spheroids from all four cell lines was significantly reduced compared to the vehicle control (Fig. 2 and Supp. Fig. 4). This phenomenon is compatible with the differential expression results, which showed a downregulation in several cell cycle progression genes in the cells treated with TVB-2640. To further define the phenotypic effects of TVB-2640, we performed a wound healing assay and a transwell invasion assay, as these permit the quantification of migration/invasion with the treatment of TVB-2640, relative to baseline controls. From the wound healing assay, we found that cell migration was impaired in the MDA-MB-231-BR cells but not parental MDA-MD-231 cells in a dose-independent manner, as migration in MDA-MB-231-BR cells was reduced with 1 μM TVB-2640, but there was no further reduction at the maximum serum concentration (*C*_max_, 10.3 μM) or the average plasma concentration (*C*_ave_, 6.24 μM) doses of TVB-2640 (Fig. 3a, b and Supp. Fig. 5a)^24^. From the transwell invasion assay, we observed that invasion was reduced in all four cell lines with the treatment of 1 μM TVB-2640 (Fig. 3c–f and Supp. Fig. 5b–e). Taken together, these experiments show that TVB-2640 alone slows the motility of breast cancer cell lines, despite the minimal impact on viability during our 5-day screening assay, reinforcing the assumption that to maximally exploit its anticancer effects, TVB-2640 should be used with a synergistic anti-proliferative drug partner. We hypothesize that FASN may play a role in not only survival in the brain microenvironment but also in the metastatic cascade steps necessary for traversion into the brain and subsequent invasion.

Our preclinical study addresses the need for more research focusing on brain-specific vulnerabilities, such as metabolic differences, investigating breast cancer BMs, especially TNBC BMs^25^. A recent study showed that the requirement for fatty acid synthesis in metastatic breast cancer is site-dependent, and especially stringent in the brain^6^. A more recent study showed that the downregulation of retinoic acid receptor responder 2 (*RARRES2*) is associated with TNBC BM through reprogramming lipid metabolism via the promotion of phosphatidylinositol kinase 3 (PI3K) signaling^26^. PIK3CA mutations and/or phosphatase and tensin homolog (PTEN) loss are common in TNBC, and activation of the PI3K pathway is strongly associated with BM and the promotion of lipid biosynthesis^27^. In our study, we reveal that the combination of SN-38 and FASN inhibitors demonstrated synergy in TNBC BM cell lines, supporting further investigation of TVB-2640 as a companion drug with SG, the current standard-of-care treatment for metastatic TNBC in the clinic. Such a postulated trial design would be greatly facilitated by the currently observed highly tolerable toxicity of TVB-2640 as monotherapy if the combination also proves tolerable. Moreover, we delineated that FASN inhibition promotes the upregulation of pro-fatty acid synthesis genes and decreases cell cycle progression and DNA damage response genes, demonstrating definitively target engagement of the drugs. The downregulation of cell cycle progression genes points to retardation in cell growth and division, and we further demonstrate this phenomenon with diminished spheroid outgrowth, migration, and invasion, which occurs despite TVB-2640’s low cytotoxicity in standard live-dead assays. More studies are needed, especially in vivo assessments of toxicity and efficacy, to fully elucidate the SN-38/SG and TVB-2640 combinations and the impact of FASN inhibition on the metastatic cascade.

## Methods

### Cell lines

MDA-MB-231-Luc/GFP (termed parental MDA-MB-231) and MDA-MB-231-BR-GFP (termed MDA-MB-231BR) cell lines were provided by Dr. Patricia Steeg at the National Cancer Institute. MDA-MB-231-Luc/GFP and MDA-MB-231-BR-GFP were authenticated using Short tandem repeat (STR) profiling (ATCC) and tested for mycoplasma. Parental MDA-MB-231 cells are available for purchase from ATCC (HTB-26).

PDO cell lines were established from resected patient tumors. The tumor tissues were obtained fresh from the operating room after patient consent and approval under the Institutional Review Board protocol at the University of Michigan (HUM00103662 and HUM00042204). Acquisition of patient material complied with all relevant ethical regulations and patient material was deidentified upon arrival at the lab. Tissue was dissociated mechanically and enzymatically using Disposable Scalpels #22 (Electron Microscopy Sciences 7204222), a gentleMACS^TM^ C-tube (Miltenyi Biotec 130-093-237), Dissociator (Miltenyi Biotec 130-093-235) and Tumor Dissociation Kit, human (Miltenyi Biotec 130-095-929). The dissociated tissue was then cultured in a Collogen I-coated T25 flask (Corning^TM^ 356484) with DMEM/F12 50:50 (Corning^TM^ 10090CV) supplemented with 10% FBS (Gibco^TM^ A5256701), 1% Antibiotic-Antimitotic (Corning^TM^ 30004CI), and 0.6% Amphotericin B (Corning^TM^ 30003CF). Cells were left to grow and were successfully passaged 1:2 at least 10 times before being used in the study. PDO cell lines were tested for mycoplasma.

### Cell culture

The MDA-MB-231-Luc/GFP and MDA-MB-231-BR-GFP cell lines were cultured in Gibco RPMI-1640 supplemented with 10% FBS (Gibco^TM^ A5256701), 1% Antibiotic-Antimitotic (Corning^TM^ 30004CI), and 0.05% Gentamicin (Gibco^TM^ 15750060). PDO-BC25 and PDO-BC25-2 were cultured with DMEM/F12 50:50 (Corning^TM^ 10090CV) supplemented with 10% FBS (Gibco A5256701), 1% Antibiotic-Antimitotic (Corning^TM^ 30004CI), and 0.6% Amphotericin B (Corning^TM^ 30003CF). For drug-screening assays, Human Plasma-like Medium (HPLM) (Gibco^TM^ A4899101) supplemented with 1% Penicillin-Streptomycin (Gibco^TM^ 15140122), 1% 2-hydroxybutyric acid (Thermo Scientific Chemicals A1863603), 0.05% Gentamicin (Gibco^TM^ 15750060), and either 10% FBS (Gibco^TM^ A5256701) or 10% Lipid-depleted FBS (Biowest LLC S148L) were used. The lipid-depleted version of HPLM was also used for the differential expression analysis experiments.

### NanoString nCounter differential expression experiments

Cells were seeded in 6-well elplasia plates (Corning^TM^ 4440) at 1.5 million cells per well on day 0 with HPLM supplied with lipid-depleted FBS. On day 0, cells were treated with TVB-2640, TVB-3166, BI-99179, or DMSO. On day 3, media was replenished with the same concentrations of TVB-2640, TVB-3166, BI-99179, or DMSO. On day 5, cells were collected, and RNA was isolated using the RNeasy Plus Mini Kit (Qiagen, Cat No. 74134). RNA was characterized using the NanoString Metabolic Pathways Panel on an nCounter Max/Flex platform, which includes a liquid handler prep station and a digital analyzer. Raw counts from the digital analyzer were analyzed using the Rosalind nCounter Data Analysis software for the Supp Fig. 2 data and nSolver Advanced Analysis software for the Fig. 1 and Supp Fig. 3 data to determine differentially expressed genes between cells treated with TVB-2640, TVB-3166, or BI-99179 versus DMSO control. Volcano plots were generated using GraphPad Prism 10.

### Drug screening

Cells were seeded in ultra-low attachment 96-well plates (S-bioTM MS-9096WZ) at a seeding density of 5000 cells/well on day 0 in HPLM supplemented with 10% FBS or lipid-depleted FBS. All drugs were serially diluted spanning 8 doses. Drugs were then diluted 1:10 in cell culture medium and then added to the cells at a 1:100 dilution on day 0, achieving a 1:1000 drug and DMSO dilution overall. For the Chou-Talalay combination screens, the final dilutions were 1:1000 for each drug and 1:500 for DMSO. All drugs were tested in triplicate. Cells were incubated at 10% CO_2_ and 37 °C. On day 5, cell viability was measured using CellTiter-Glo 3D (Promega, G9681) reagent and a BioTek Synergy H1 Microplate Reader (Agilent). Dose-response curves were plotted in GraphPad Prism 10 using a four-parameter nonlinear regression model, fitted into the equation *Y* = Bottom + (Top – Bottom)/(1 + 10^(Log10(IC50)^ ^– ^*X*) * Hill Slope) where *Y* = % cell viability relative to the vehicle and *X* = Log10(Molar Concentration). The fitted curves were constrained to Bottom > 0 and Top = 100. CompuSyn software (ComboSyn, Inc., Paramus, NJ) was used to analyze the drug combination data following the Chou-Talalay method^19,20,28^.

### Spheroid outgrowth assays

Cells were seeded in ultra-low attachment 96-well plates (CorningTM 4520) at a seeding density of 3000 cells/well on day 0 in HPLM supplemented with 10% FBS, followed by incubation in 10% CO_2_ and 37 °C. On day 1, TVB-2640 was added at a concentration of 1 µM to the treated wells and the control wells received an equivalent volume of DMSO, and Growth Factor Reduced Matrigel (CorningTM 356231) was added at a final concentration of 25% (v/v). The plate was then incubated in a BioSpa 8 Automatic Incubator coupled with a Citation 5 Cell Imaging Multi-Mode Reader and imaged every 6 h over 9 days. Image J was used to measure the areas of the spheroids at each time point and GraphPad Prism 10 was used to plot relative outgrowth curves. Area under the curve (AUC) calculations were performed for each replicate using the Area Under the Curve function in Graph Pad Prism 10.

### Transwell invasion assays

Cells were seeded in 2D culture in the presence of DMSO or 1 µM TVB-2640 in HPLM supplemented with 10% FBS and incubated for 1 day in a 10% CO_2_ and 37 °C incubator. Cells were then dissociated with trypsin, counted, and seeded in Corning Matrigel Invasion Chamber (CorningTM 354480) 8.0 µm inserts with DMSO or 1 µM TVB-2640 in 3 replicates and incubated for 1 day in a 10% CO_2_ and 37 °C incubator. MDA-MB-231-BR was seeded at 1.5 × 10^5^ cells per insert while MDA-MB-231, PDO-BC25, and PDO-BC25-2 were seeded at 5.0 × 10^4^ cells per insert. Cells were seeded with 500 µL serum-free HPLM in the upper chamber and 750 µL HPLM supplemented with 10% lipid-depleted FBS was added in the lower chamber. Prior to seeding the cells, the inserts were rehydrated by adding HPLM to both chambers and incubated for 2 h. Inserts were then removed and cells in the upper chamber were swabbed out using a cotton tip applicator. Each insert was fixed with 70% ethanol for 15 min, set to dry, then stained with 1% toluidine blue for 10 min and washed 3 times with water. After the inserts dried, they were imaged using an EVOS M5000 microscope (ThermoFisher AMF5000). Four regions per insert were imaged and cells were counted using the cell counter tool in Image J and plotted in Graphpad Prism 10.

### Wound healing assays

Cells were seeded in a Corning Costar flat-bottom 96-well plate (CorningTM 3997) at 20,000 cells/well with HPLM supplemented with 10% FBS and treated with DMSO or TVB-2640 at 1 µM, average serum concentration (*C*_avg_) 6.24 µM, or peak serum concentration (*C*_max_) 10.3 µM. After 1 day, cells were scratched with a P200 micropipette tip, washed twice with PBS, and supplemented with fresh media containing TVB-2640 or DMSO. The cells were then imaged every 6 h over 30 h using a BioSpa 8 Automatic Incubator coupled with a Citation 5 Cell Imaging Multi-Mode Reader. Wound closure at each time point was calculated using an image J plugin^29^. Regions of interest were drawn on clean scratches so that only the scratched area was counted by the plugin. Parameters were adjusted as needed to quantify only the scratched area, but the average parameters were: Variance window ratio: 5; Threshold value: 30; and Percentage of saturated pixels: 0.1. Results were plotted in GraphPad Prism 10.

### Reagents

Compounds were purchased from Selleckchem (Houston, TX) and MedChemExpress (Monmouth Junction, NJ). All compounds were diluted in DMSO (Sigma-Aldrich, D2650).

### Statistical analysis

For the Nanostring DE experiments, a *t*-test was performed to calculate *p*-values for the TVB-2640, TVB-3166, or BI-99179 treatments versus DMSO control, and the Benjamini–Hochberg (BH) method was used to adjust for the false discovery rate and calculate adjusted *p*-values (p*). Batch number and cell line were set as confounders when analyzing all cell lines together, and batch number alone was set as a confounder when analyzing each cell line individually. For the data in Supp Fig. 2, the *N* = 2 biological replicates per treatment per cell line were included and the Rosalind nCounter Data Analysis software was used to perform the statistical analysis. For the data in Fig.1 and Supp. Fig. 3, *N* = 4 biological replicates per treatment per cell line were included and the Nanostring nSolver Advanced Analysis software was used to perform the statistical analysis. For the spheroid outgrowth assay, multiple unpaired *t*-tests were performed for each data point in GraphPad Prism 10 with *N* = 6 biological replicates included per treatment per cell line. For the spheroid outgrowth experiment’s area under the curve (AUC) calculations, an unpaired non-parametric Mann–Whitney test was performed with *N* = 6 biological replicates per treatment per cell line in GraphPad Prism 10. For the transwell migration and wound healing assays, statistics were calculated using multiple unpaired *t*-tests comparing experimental conditions to DMSO control.

### DNA sequencing

DNA from both parent tumors and cell lines of PDO-BC25 and PDO-BC25-2 were sequenced via next-generation sequencing using the Oncomine Comprehensive Panel (OCP), a previously published targeted pan-cancer assay^30,31^. The OCP targets entire coding regions of tumor suppressor genes and regions of oncogenes known to give rise to the gain of function mutations if mutated. Moreover, OCP targets genomic regions for a subset of genes with recurrent copy number alterations across the gene body to detect gains and losses. Only deep deletions (2 copy loss) and high-level amplifications (2 or more) were prioritized in this study. Both the parent tumors and cell lines of both samples revealed the same variants and copy number alterations (Supp. Table 4). The variants detected were TP53 p.Y220H, PIK3CA p.H1047R, and RAD51 p.E77fs. The copy number alterations detected were deep deletions (two-copy loss) in each of MTAP, CDKN2A, and CDKN2B.

### G-band karyotyping

PDO-BC25 and PDO-BC25-2 cells were each seeded in a T25 flask and shipped live to Cell Line Genetics (Madison, WI), following the company’s instructions. Cytogenetic analysis was performed on ten G-banded cells at metaphase for each cell line, revealing abnormal human triploid karyotypes (Supp. Fig. 6). PDO-BC25 demonstrated chromosomal counts ranging from 55 to 65. Aberrations seen in two or more cells were a deletion in the short arm of chromosome 4 between bands p14 and p16, a deletion on the long arm of chromosome 6 at band q12, additional genetic material of unknown origin translocated to the short arm of chromosome 8 at band p11.2, a deletion on the short arm of chromosome 8 at band p12, up to two copies of additional genetic material of unknown origin translocated to the short arm of chromosome 9 at band p11, a deletion on the long arm of chromosome 12 between bands q13 and q21, an unbalanced translocation between the long arm of chromosome 12 at band q22 and the long arm of chromosome 1 at band q41, additional genetic material of unknown origin translocated to the short arm of chromosome 13 at band p12, an unbalanced whole-arm translocation between the long arm of chromosome 13 at band q10 and the long arm of chromosome 15 at band q10, a deletion on the long arm of chromosome 14 between bands q11.2 and q13, an insertion of unknown genetic material into the long arm of chromosome 15 at band q22, up to two copies of additional genetic material of unknown origin translocated to the long arm of chromosome 22 at band q11.2, gain of 1–6 marker chromosomes of unknown origin with up to two copies of marker 1 chromosome, gain of up to two copies of chromosome 8, gain of one copy of chromosomes 14 and 21, loss of up to two copies of chromosome 9, loss of one copy of a sex chromosome, and loss of one copy of chromosomes 1, 2, 3, 4, 5, 11, 13, 16, 17, 18, 19, 20, and 22. PDO-BC25-2 demonstrated chromosomal counts ranging from 57 to 71. Aberrations seen in two or more cells were additional genetic material of unknown origin translocated to the short arm of chromosome X at band p22.1, additional genetic material of unknown origin translocated to the long arm of chromosome X at band q22, deletion of chromosome 1 in the long arm at band q24, additional genetic material of unknown origin translocated to the short arm of chromosome 2 at band p13, deletion of chromosome 2 in the long arm at band q31, deletion of chromosome 4 in the short arm between bands p14 and p16, an unbalanced translocation between the long arm of chromosome 5 at band q35 and the long arm of chromosome 8 at band q13, deletion of chromosome 6 in the long arm at band q12, additional genetic material of unknown origin translocated to the short arm of chromosome 8 at band p11.2, additional genetic material of unknown origin translocated to the short arm of two copies of chromosome 9 at band p11, additional genetic material of unknown origin translocated to the long arm of chromosome 12 at band q13, deletion of chromosome 12 in the long arm between bands q13 and q21, additional genetic material of unknown origin translocated to the short arm of two copies of chromosome 13 at band p12, deletion of chromosome 14 in the long arm between bands q11.2 and q13, additional genetic material of unknown origin translocated to the short arm of chromosome 15 at band p12, additional genetic material of unknown origin inserted into the long arm of chromosome 15 at band q22, additional genetic material of unknown origin translocated to the long arm of chromosome 19 at band q13.3, additional genetic material of unknown origin translocated to the long arm of chromosome 21 at band q21, additional genetic material of unknown origin translocated to the long arm of chromosome 22 at band q11.2, gain of one copy of chromosomes 3, 4, 12, 19, and 21, gain of two copies of chromosome 8, loss of one copy of chromosomes X, 1, 5, 10, 11, 13, 14, 15, 16, 17, 18, 20, and 22, loss of two copies of chromosome 9, and gain of up to nine marker chromosomes.

### Reporting summary

Further information on research design is available in the Nature Research Reporting Summary linked to this article.

### Supplementary information

Supplemental Figures and Tables

Reporting Summary

## Data Availability

All data generated or analyzed during this study are included in this published article or are available from the corresponding author on reasonable request.

## Code availability

This paper does not report the original code. Any additional information required to reanalyze the data reported in this work is available from the corresponding author upon reasonable request.
