# Supplementary material for: Targeting fatty acid synthase in preclinical models of TNBC brain metastases synergizes with SN-38 and impairs invasion
Source: NPJ Breast Cancer. 2024 Jun 10;10:43. doi: 10.1038/s41523-024-00656-0 (PMC11164988; doi:10.1038/s41523-024-00656-0)
Supplement: Supplementary file 1 — Supplemental Figures and Tables [file 41523_2024_656_MOESM1_ESM.pdf]

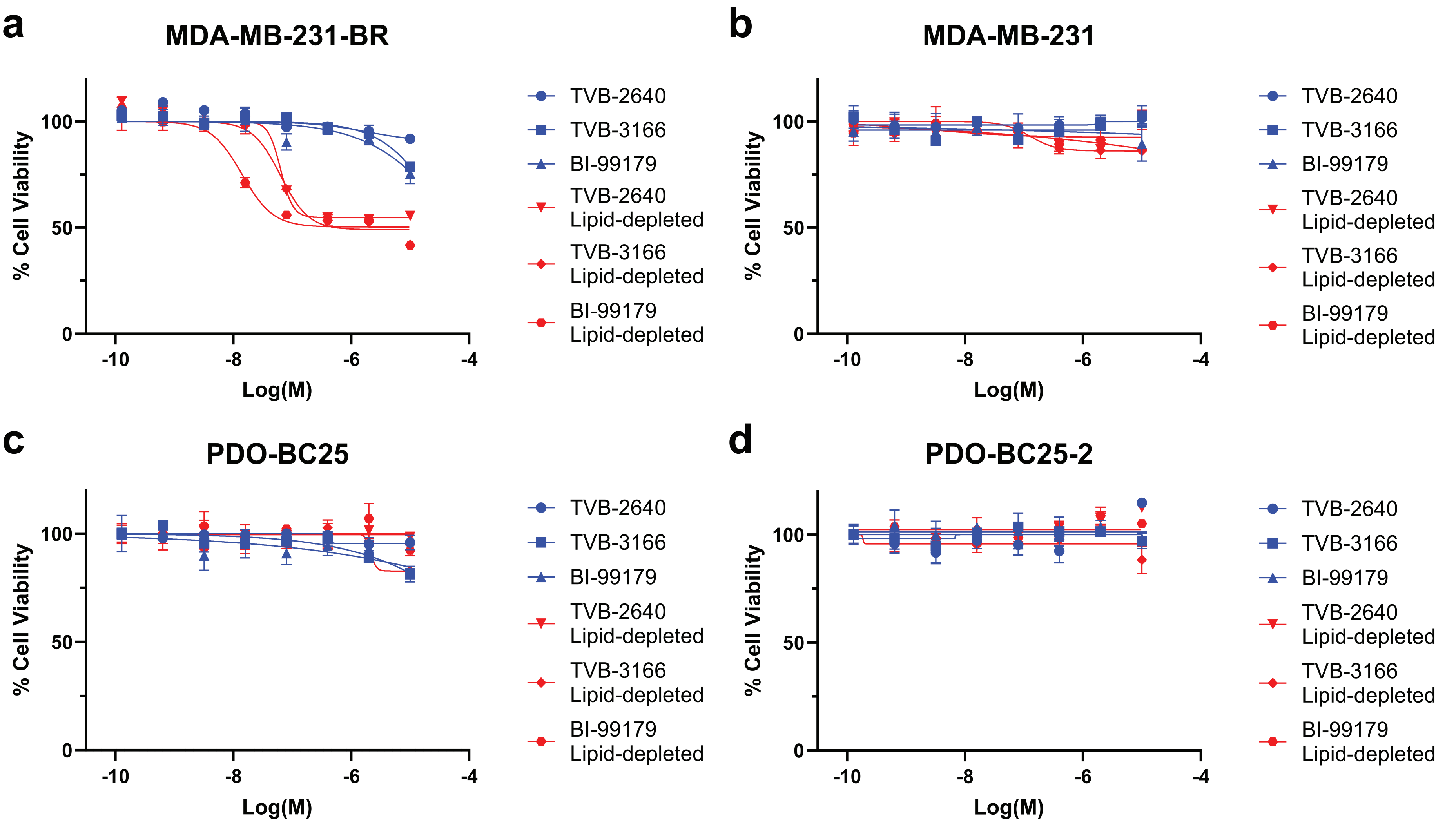


**Supplemental Figure 1: FASN inhibitors demonstrate low toxicity in TNBC BM cell lines.** Dose-response curves were generated for FASN inhibitors screened in cells cultured with HPLM supplemented with FBS or lipid-depleted FBS in 3D culture for **(a)** MDA-MB-231-BR; **(b)** MDA-MB-231; **(c)** PDO-BC25; and **(d)** PDO-BC-25-2. N = 3 replicates. Error bars indicate SD.

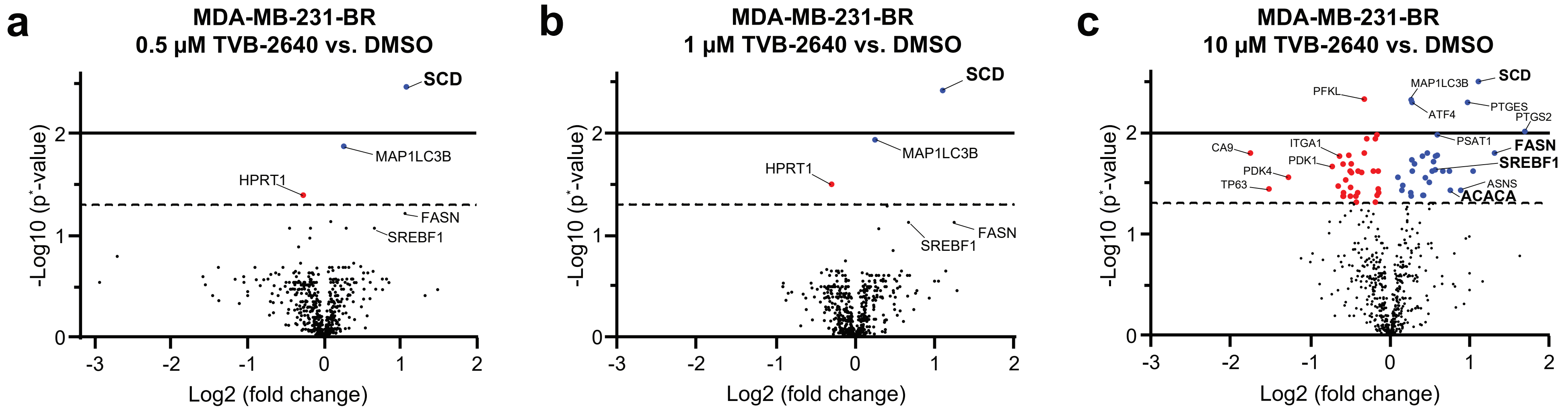


**Supplemental Figure 2: FASN inhibition with TVB-2640 shows target engagement in MDA-MB-231-BR cells.** Volcano plots were generated showing DE analysis for a panel of metabolic genes using RNA collected from MDA-MB-231-BR spheroids treated with 0.5 µM **(a)**, 1 µM **(b)**, or 10 µM **(c)** TVB-2640 vs. DMSO control. N = 2 biological replicates per treatment. Dashed line indicates p adjusted = 0.05 and solid line indicates p adjusted = 0.01 by the Benjamini-Hochberg method.

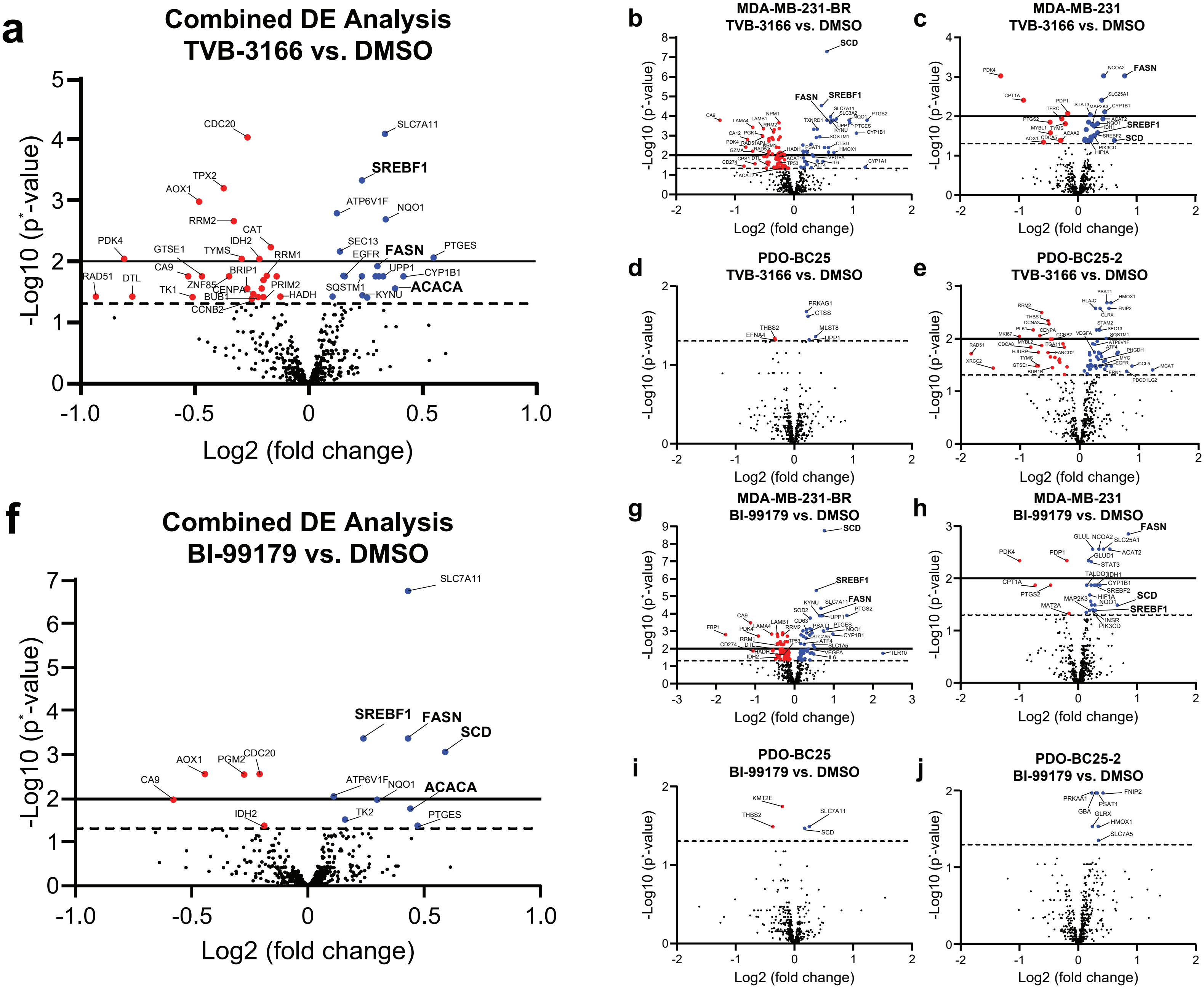


**Supplemental Figure 3: FASN inhibition with TVB-3166 and BI-99179 demonstrates evidence of target engagement in TNBC BM cell lines.** Volcano plots were generated showing DE analysis for a panel of metabolic genes using RNA collected from spheroids treated with 1 μM TVB-3166 or BI-99179 vs. DMSO control in HPLM supplemented with lipid-depleted FBS in 3D culture. 1 μM TVB-3166 vs. DMSO was compared for: **(a)** Combined DE analysis for all four cell lines; **(b)** MDA-MB-231-BR; **(c)** MDA-MB-231; **(d)** PDO-BC25; and **(e)** PDO-BC25-2. 1 μM BI-99179 vs. DMSO was compared for: **(f)** Combined DE analysis for all four cell lines; **(g)** MDA-MB-231-BR; **(h)** MDA-MB-231; **(i)** PDO-BC25; and **(j)** PDO-BC25-2. N = 4 biological replicates per cell line per treatment. Dashed line indicates p adjusted = 0.05 and solid line indicates p adjusted = 0.01 by the Benjamini-Hochberg method.

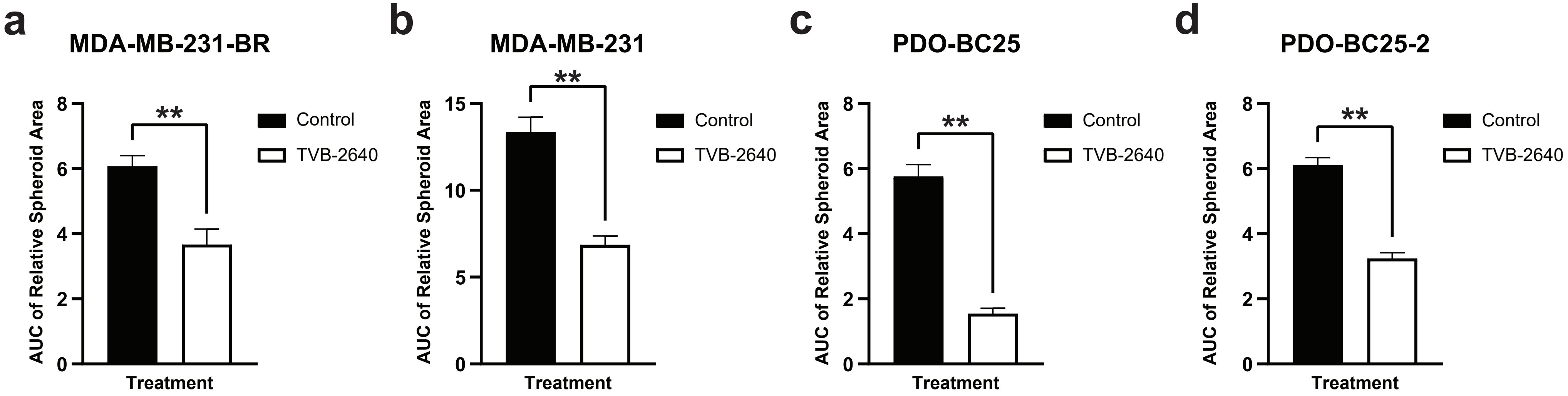


**Supplemental Figure 4: Area under the curve measurements of the spheroid outgrowth curves from Figure 2 for (a)** MDA-MB-231-BR; **(b)** MDA-MB-231; **(c)** PDO-BC25; and **(d)** PDO-BC25-2. N = 6 replicates. **p adjusted < 0.01 by Mann-Whitney test. Error bars indicate SEM.

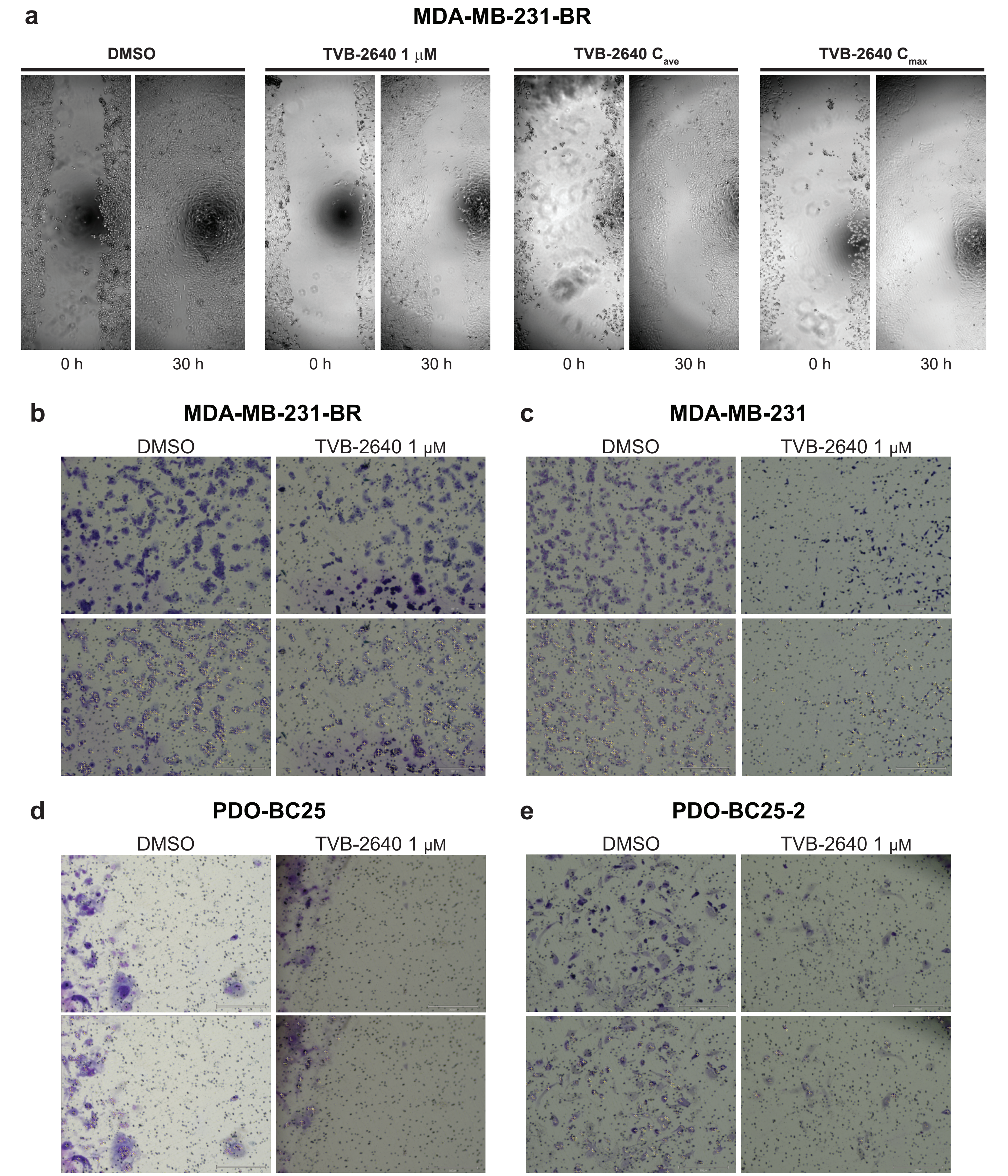


**Supplemental Figure 5: Representative images of cells from the wound healing migration and transwell invasion assays in Figure 3. (a)** Images of MDA-MB-231-BR in the wound healing migration assay at 0 and 30 hour time points treated with DMSO or 1 μM, Cave, or Cmax TVB-2640. Images of cells treated with DMSO or 1 μM TVB-2640 that successfully invaded through the barrier in the transwell invasion assay for: **(b)** MDA-MB-231-BR; **(c)** MDA-MB-231; **(d)** PDO-BC25; and **(e)** PDO-BC25-2. Lower images show the counted cells from the upper images. Scale bars indicate 300 μm.

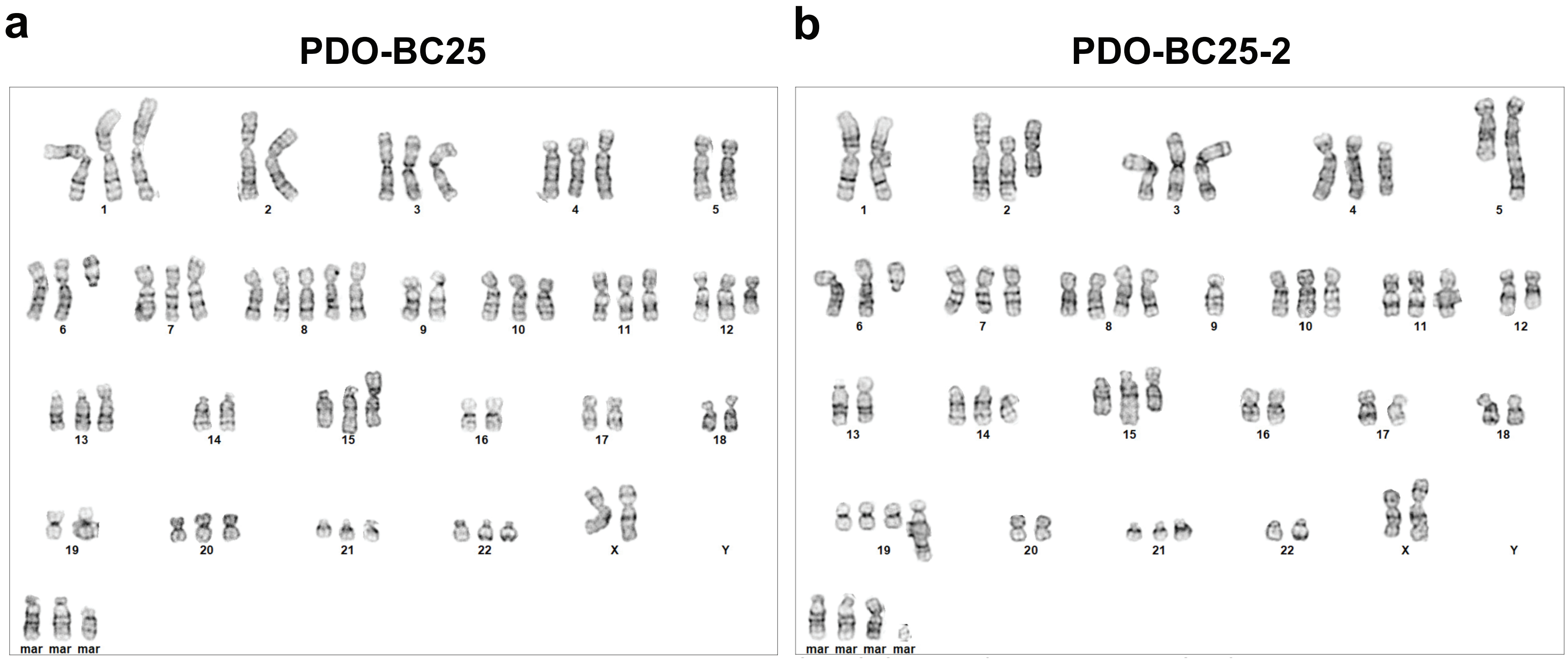


**Supplemental Figure 6: PDO-BC25 and PDO-BC25-2 cell lines possess abnormal triploid karyotypes.** Cytogenetic analysis was performed on ten G-banded cells at metaphase for each cell line: **(a)** PDO-BC25 and **(b)** PDO-BC25-2.

**Supplemental Table 1:** Clinical background of the patient with the TNBC BM tumors used to generate the PDO-BC25 and PDO-BC25-2 Cell Lines

<table>
<tr><th>Cell lines</th><th>ER/PR/HER2 status</th><th>Age at tissue collection</th><th>Race/Ethnicity</th><th>Prior therapy before tissue collection</th><th>Germline BRCA status</th><th>PI3K pathway-related alterations (clinical testing)</th><th>PD-L1 status</th></tr>
<tr><td>PDO-BC25</td><td rowspan="2">ER/PR/HER2 negative</td><td rowspan="2">61</td><td rowspan="2">Caucasion</td><td>Doxorubicin, cyclophosphamide, Nab-paclitaxel, capecitabine, bevacizumab, gemcitabine, cisplatin, paclitaxel, eribulin</td><td rowspan="2">Negative</td><td rowspan="2">PIK3CA mutation and PTEN homozygous deletion</td><td rowspan="2">Positive (CPS>50)</td></tr>
<tr><td>PDO-BC25-2</td><td>As above + gemcitabine and pembrolizumab after first collection</td></tr>
</table>

**Supplemental Table 2:** Combination index values at therapeutically relevant doses (ED50 and ED75) of SN-38 in combination with the FASN inhibitors TVB-3166 and BI-99179

| Cell Line \ Drug Combination | SN-38 + TVB-3166 | | | | SN-38 + BI-99179 | | | |
|---|---|---|---|---|---|---|---|---|
| | HPLM | | Lipid-depleted HPLM | | HPLM | | Lipid-depleted HPLM | |
| | ED50 | ED75 | ED50 | ED75 | ED50 | ED75 | ED50 | ED75 |
| **MDA-MB-231** | 6.49E+24 | 3.52E+35 | 1.05 | 1.30 | 1.69E+13 | 9.63E+19 | 1.24 | 1.63 |
| **MDA-MB-231-BR** | 0.40 | 0.99 | 0.25 | 0.59 | 0.45 | 1.45 | 0.2 | 1.04 |
| **PDO-BC25** | 0.69 | 0.38 | 0.68 | 0.68 | 1.08 | 1.04 | 0.64 | 0.24 |
| **PDO-BC-25-2** | 0.36 | 0.24 | 0.43 | 0.40 | 0.07 | 0.01 | 0.72 | 0.17 |

**Supplementary Table 3. Significant DE genes with FASN inhibition.**

| All 4 combined TVB-2640 vs. DMSO | | | |
|---|---|---|---|
| **Gene** | **Log2 Fold Change** | **p* value** | **Gene Set** |
| **SREBF1** | 0.337 | 2.21E-08 | AMPK, Transcriptional Regulation |
| **FASN** | 0.493 | 2.46E-06 | AMPK, Transcriptional Regulation, Fatty Acid Synthesis |
| **AOX1** | -0.487 | 2.18E-04 | Amino Acid Synthesis, Cytokine & Chemokine Signaling, TryptophanKynurenine Metabolism, Vitamin & Cofactor Metabolism |
| **RRM1** | -0.238 | 2.18E-04 | Nucleotide Synthesis |
| **CDC20** | -0.216 | 2.18E-04 | Antigen Presentation, Cell Cycle |
| **SCD** | 0.561 | 2.18E-04 | AMPK, Fatty Acid Synthesis |
| **GCLC** | 0.168 | 1.33E-03 | Amino Acid Synthesis, Reactive Oxygen Response |
| **BRIP1** | -0.327 | 2.12E-03 | Cell Cycle, DNA Damage Repair |
| **RRM2** | -0.284 | 2.58E-03 | Cell Cycle, Nucleotide Synthesis |
| **TPX2** | -0.287 | 2.81E-03 | Cell Cycle |
| **CAT** | -0.146 | 4.96E-03 | Reactive Oxygen Response, TryptophanKynurenine Metabolism |
| **CENPA** | -0.277 | 5.26E-03 | Cell Cycle |
| **UBE2C** | -0.208 | 5.85E-03 | Antigen Presentation, Cell Cycle |
| **PDK4** | -0.727 | 6.72E-03 | Mitochondrial Respiration |
| **NQO1** | 0.268 | 6.72E-03 | KEAP1NRF2 Pathway, Reactive Oxygen Response |
| **PRIM2** | -0.222 | 7.47E-03 | Cell Cycle, Nucleotide Synthesis |
| **CCNA2** | -0.242 | 8.79E-03 | AMPK, Cell Cycle, DNA Damage Repair, Myc |
| **PGD** | 0.145 | 9.25E-03 | Pentose Phosphate Pathway |
| **MYBL2** | -0.756 | 1.08E-02 | Cell Cycle, Transcriptional Regulation |
| **SQSTM1** | 0.227 | 1.23E-02 | Cytokine & Chemokine Signaling |
| **DTL** | -0.797 | 1.29E-02 | DNA Damage Repair |
| **MKI67** | -0.398 | 1.29E-02 | Cell Cycle |
| **BUB1** | -0.23 | 1.29E-02 | Cell Cycle |
| **ATP6V1F** | 0.0881 | 1.29E-02 | Mitochondrial Respiration, mTOR |
| **KYNU** | 0.25 | 1.29E-02 | TryptophanKynurenine Metabolism |
| **SLC3A2** | 0.277 | 1.29E-02 | Amino Acid Transporters, mTOR |
| **KPNA2** | -0.122 | 1.46E-02 | Cytokine & Chemokine Signaling, DNA Damage Repair |

| | | | |
|---|---|---|---|
| **SLC7A11** | 0.177 | 1.56E-02 | Amino Acid Transporters, Reactive Oxygen Response |
| **NDUFA12** | -0.105 | 1.60E-02 | Mitochondrial Respiration, Reactive Oxygen Response |
| **PCLAF** | -0.398 | 1.75E-02 | DNA Damage Repair |
| **NCAPH** | -0.345 | 1.89E-02 | Cell Cycle |
| **UPP1** | 0.291 | 1.94E-02 | Nucleotide Salvage, Nucleotide Synthesis |
| **TKT** | 0.197 | 2.27E-02 | Pentose Phosphate Pathway |
| **EXO1** | -0.394 | 2.33E-02 | Cell Cycle, DNA Damage Repair |
| **CA9** | -0.431 | 2.97E-02 | Hypoxia |
| **CPT1A** | -0.278 | 3.07E-02 | AMPK, Fatty Acid Oxidation |
| **TYMS** | -0.205 | 3.20E-02 | Cell Cycle, Nucleotide Synthesis |
| **CCNB2** | -0.229 | 3.31E-02 | Cell Cycle |
| **EEA1** | -0.106 | 3.31E-02 | Endocytosis, TLR Signaling |
| **IDH2** | -0.154 | 3.38E-02 | IDH12 Activity, Mitochondrial Respiration |
| **XRCC2** | -0.303 | 3.69E-02 | DNA Damage Repair |
| **ZNF91** | -0.198 | 4.27E-02 | Transcriptional Regulation |
| **HADH** | -0.106 | 4.27E-02 | Amino Acid Synthesis, Fatty Acid Oxidation, TryptophanKynurenine Metabolism |
| **PGM2** | -0.166 | 4.36E-02 | Glycolysis, Nucleotide Synthesis, Pentose Phosphate Pathway |
| **GPX4** | 0.0683 | 4.36E-02 | Reactive Oxygen Response |
| **TF** | 0.405 | 4.36E-02 | Endocytosis |
| **TK2** | 0.12 | 4.44E-02 | Nucleotide Salvage, Nucleotide Synthesis |

| MDA-MB-231-BR TVB-2640 vs. DMSO | | | |
|---|---|---|---|
| **Gene** | **Log2 Fold Change** | **p* value** | **Gene Set** |
| **SCD** | 0.727 | 3.03E-09 | AMPK, Fatty Acid Synthesis |
| **SREBF1** | 0.525 | 8.69E-06 | AMPK, Transcriptional Regulation, Fatty Acid Synthesis |
| **FASN** | 0.796 | 6.87E-05 | AMPK, Fatty Acid Synthesis, Myc, Vitamin & Cofactor Metabolism |
| **PTGS2** | 1.34 | 1.29E-04 | Cytokine & Chemokine Signaling, NF-kB, Reactive Oxygen Response, Vitamin & Cofactor Metabolism |
| **SLC3A2** | 0.699 | 1.29E-04 | Amino Acid Transporters, mTOR |
| **KYNU** | 0.672 | 1.29E-04 | TryptophanKynurenine Metabolism |
| **UPP1** | 0.68 | 1.83E-04 | Nucleotide Salvage, Nucleotide Synthesis |
| **NPM1** | -0.266 | 2.20E-04 | Cell Cycle, Myc |
| **SLC7A11** | 0.514 | 2.46E-04 | Amino Acid Transporters, Reactive Oxygen Response |
| **CA9** | -1.14 | 2.98E-04 | Hypoxia |
| **PTGES** | 0.88 | 5.12E-04 | Glutamine Metabolism |
| **SOD2** | 0.348 | 5.12E-04 | Cytokine & Chemokine Signaling, Mitochondrial Respiration |
| **PEBP1** | -0.27 | 6.64E-04 | Reactive Oxygen Response |
| **RRM2** | -0.362 | 6.64E-04 | Cell Cycle, Nucleotide Synthesis |
| **NQO1** | 0.773 | 6.86E-04 | KEAP1NRF2 Pathway, Reactive Oxygen Response |
| **LTA4H** | -0.31 | 7.60E-04 | Amino Acid Synthesis |
| **BRIP1** | -0.605 | 7.66E-04 | Cell Cycle, DNA Damage Repair |
| **TXNRD1** | 0.372 | 7.86E-04 | p53 Pathway |
| **CYP1B1** | 1.06 | 8.66E-04 | TryptophanKynurenine Metabolism |
| **IMPDH2** | -0.223 | 9.50E-04 | Nucleotide Synthesis |
| **LAMB1** | -0.454 | 1.19E-03 | PI3K |
| **GAPDH** | -0.291 | 1.61E-03 | Glycolysis |
| **SQSTM1** | 0.42 | 1.81E-03 | Cytokine & Chemokine Signaling |
| **DCK** | -0.24 | 2.59E-03 | Nucleotide Salvage, Nucleotide Synthesis |
| **LDHB** | -0.256 | 2.90E-03 | Amino Acid Synthesis, Glycolysis, Mitochondrial Respiration |
| **GUSB** | -0.279 | 3.00E-03 | Lysosomal Degradation |
| **PDK4** | -0.876 | 3.00E-03 | Mitochondrial Respiration |

| | | | |
|---|---|---|---|
| VEGFA | 0.435 | 3.26E-03 | Cytokine & Chemokine Signaling, Hypoxia, MAPK, PI3K |
| PRIM1 | -0.379 | 3.71E-03 | Cell Cycle, Nucleotide Synthesis |
| SLC7A5 | 0.276 | 3.91E-03 | Amino Acid Transporters, mTOR |
| PSAT1 | 0.25 | 3.91E-03 | Amino Acid Synthesis, Glutamine Metabolism |
| MAP1LC3E | 0.215 | 3.91E-03 | Autophagy |
| GMPR2 | -0.233 | 3.91E-03 | Nucleotide Salvage, Nucleotide Synthesis |
| MSH2 | -0.26 | 3.91E-03 | DNA Damage Repair |
| HPRT1 | -0.275 | 3.91E-03 | Nucleotide Salvage, Nucleotide Synthesis |
| ACAA2 | -0.299 | 3.91E-03 | Amino Acid Synthesis, Fatty Acid Oxidation |
| NCAPH | -0.391 | 3.91E-03 | Cell Cycle |
| PGK1 | -0.434 | 3.91E-03 | Glycolysis |
| FBP1 | -1.52 | 3.91E-03 | AMPK, Glycolysis, Pentose Phosphate Pathway |
| CAT | -0.306 | 4.49E-03 | Reactive Oxygen Response, TryptophanKynurenine Metabolism |
| ACAT1 | -0.327 | 4.63E-03 | Amino Acid Synthesis, Fatty Acid Oxidation, TryptophanKynurenine Metabolism |
| RAD51AP1 | -0.411 | 5.40E-03 | DNA Damage Repair |
| GLUD1 | -0.18 | 5.72E-03 | Amino Acid Synthesis, Arginine Metabolism, Glutamine Metabolism, Mitochondrial Respiration |
| HADH | -0.296 | 5.86E-03 | Amino Acid Synthesis, Fatty Acid Oxidation, TryptophanKynurenine Metabolism |
| UBE2C | -0.396 | 6.14E-03 | Antigen Presentation, Cell Cycle |
| ECHS1 | -0.166 | 6.25E-03 | Amino Acid Synthesis, Fatty Acid Oxidation, TryptophanKynurenine Metabolism |
| FABP5 | -0.319 | 6.25E-03 | Fatty Acid Synthesis |
| GLUL | -0.271 | 6.91E-03 | Amino Acid Synthesis, Arginine Metabolism, Glutamine Metabolism |
| SLC1A5 | 0.468 | 7.00E-03 | Amino Acid Transporters |
| IL6 | 0.608 | 7.02E-03 | Cytokine & Chemokine Signaling, PI3K |
| TLR4 | 0.285 | 7.02E-03 | Antigen Presentation, NF-kB, PI3K, TLR Signaling |
| COPS6 | -0.238 | 7.02E-03 | DNA Damage Repair, Endocytosis |
| CCNB2 | -0.269 | 7.29E-03 | Cell Cycle |
| RRM1 | -0.402 | 7.51E-03 | Nucleotide Synthesis |
| NFE2L2 | 0.125 | 7.80E-03 | IDH12 Activity, KEAP1NRF2 Pathway, Transcriptional Regulation |
| NME2 | -0.156 | 7.80E-03 | Myc, Nucleotide Synthesis |
| NDUFA12 | -0.203 | 7.80E-03 | Mitochondrial Respiration, Reactive Oxygen Response |
| TYMS | -0.27 | 7.80E-03 | Cell Cycle, Nucleotide Synthesis |
| GTSE1 | -0.323 | 7.80E-03 | Cell Cycle |
| BUB1 | -0.394 | 7.80E-03 | Cell Cycle |
| PDK1 | -0.404 | 8.70E-03 | Mitochondrial Respiration |
| CTSD | 0.495 | 8.97E-03 | Antigen Presentation, Autophagy, Lysosomal Degradation |

| | | | |
|---|---|---|---|
| ADK | -0.223 | 8.97E-03 | Nucleotide Salvage, Nucleotide Synthesis |
| TK1 | -0.3 | 9.10E-03 | Cell Cycle, Nucleotide Salvage, Nucleotide Synthesis |
| LAMA4 | -0.397 | 1.01E-02 | PI3K |
| CD63 | 0.185 | 1.05E-02 | Lysosomal Degradation |
| PGM2 | -0.254 | 1.05E-02 | Glycolysis, Nucleotide Synthesis, Pentose Phosphate Pathway |
| YWHAZ | -0.156 | 1.07E-02 | Cell Cycle, Cytokine & Chemokine Signaling, p53 Pathway, PI3K |
| RPIA | -0.283 | 1.07E-02 | Pentose Phosphate Pathway |
| TK2 | 0.256 | 1.08E-02 | Nucleotide Salvage, Nucleotide Synthesis |
| GNG12 | -0.138 | 1.08E-02 | Cytokine & Chemokine Signaling, MAPK, PI3K |
| TKT | 0.142 | 1.12E-02 | Pentose Phosphate Pathway |
| ERN1 | 0.429 | 1.21E-02 | Autophagy |
| ITGB5 | 0.114 | 1.23E-02 | Antigen Presentation, PI3K |
| NDUFB4 | -0.125 | 1.27E-02 | Mitochondrial Respiration, Reactive Oxygen Response |
| SOS2 | 0.257 | 1.40E-02 | Cytokine & Chemokine Signaling, MAPK, mTOR, PI3K, TCR & Costimulatory Signaling |
| EPC1 | 0.222 | 1.58E-02 | Epigenetic Regulation |
| CA12 | -0.521 | 1.60E-02 | Reactive Oxygen Response |
| MYC | -0.126 | 1.75E-02 | Cell Cycle, Cytokine & Chemokine Signaling, MAPK, Myc, PI3K, Transcriptional Regulation |
| COX5A | -0.167 | 1.75E-02 | Mitochondrial Respiration, p53 Pathway |
| BUB1B | -0.239 | 1.87E-02 | Cell Cycle |
| CCNA2 | -0.246 | 1.87E-02 | AMPK, Cell Cycle, DNA Damage Repair, Myc |
| TLR10 | 2.22 | 1.97E-02 | TLR Signaling |
| CMKLR1 | 0.244 | 1.97E-02 | MAPK, PI3K |
| KIF2C | -0.277 | 1.97E-02 | Antigen Presentation, Cell Cycle |
| SOD3 | -0.279 | 2.44E-02 | Reactive Oxygen Response |
| CLOCK | -0.279 | 2.58E-02 | Epigenetic Regulation, Transcriptional Regulation |
| ALDOA | -0.177 | 2.75E-02 | Glycolysis, Pentose Phosphate Pathway |
| PRPS1 | -0.265 | 2.85E-02 | Nucleotide Synthesis, Pentose Phosphate Pathway |
| COX6B1 | -0.124 | 3.07E-02 | Mitochondrial Respiration, p53 Pathway |
| UBE2T | -0.242 | 3.07E-02 | DNA Damage Repair |
| AFMID | -0.365 | 3.07E-02 | TryptophanKynurenine Metabolism |
| CS | -0.111 | 3.19E-02 | Mitochondrial Respiration |
| ATF4 | 0.171 | 3.22E-02 | MAPK, PI3K, Transcriptional Regulation |
| GZMA | -0.498 | 3.22E-02 | |
| HK2 | -0.243 | 3.25E-02 | Glycolysis |
| RICTOR | 0.105 | 3.59E-02 | mTOR |
| CPS1 | -0.619 | 3.59E-02 | Amino Acid Synthesis, Arginine Metabolism |
| LEPR | -0.233 | 3.68E-02 | AMPK, Cytokine & Chemokine Signaling |
| ENO1 | -0.232 | 3.74E-02 | Glycolysis, Myc |

| | | | |
|---|---|---|---|
| **PRKAA2** | -0.346 | 3.75E-02 | AMPK, Autophagy, Fatty Acid Oxidation, Mitochondrial Respiration, mTOR, p53 Pathway, PI3K |
| **THBS1** | 0.407 | 3.79E-02 | Myc, PI3K |
| **ATG101** | 0.176 | 3.98E-02 | Autophagy |
| **PDK3** | -0.259 | 3.98E-02 | Mitochondrial Respiration |
| **FH** | -0.164 | 4.04E-02 | IDH12 Activity, Mitochondrial Respiration |
| **FNIP1** | 0.197 | 4.14E-02 | mTOR |
| **CDC20** | -0.247 | 4.14E-02 | Antigen Presentation, Cell Cycle |
| **TBK1** | 0.116 | 4.28E-02 | Cytokine & Chemokine Signaling, TLR Signaling |
| **CHMP2A** | -0.194 | 4.29E-02 | Autophagy, Endocytosis |
| **CD274** | -0.801 | 4.39E-02 | TCR & Costimulatory Signaling |
| **NDUFA2** | -0.327 | 4.79E-02 | Mitochondrial Respiration |

| MDA-MB-231 TVB-2640 vs. DMSO | | | |
|---|---|---|---|
| **Gene** | **Log2 Fold Change** | **p* value** | **Gene Set** |
| **FASN** | 0.803 | 7.68E-04 | AMPK, Fatty Acid Synthesis, Myc, Vitamin & Cofactor Metabolism |
| **PDK4** | -1.12 | 1.98E-03 | Mitochondrial Respiration |
| **ACAT2** | 0.515 | 2.28E-03 | Amino Acid Synthesis, Fatty Acid Oxidation, TryptophanKynurenine Metabolism |
| **NQO1** | 0.391 | 5.27E-03 | KEAP1NRF2 Pathway, Reactive Oxygen Response |
| **SREBF1** | 0.418 | 6.18E-03 | AMPK, Transcriptional Regulation, Fatty Acid Synthesis |
| **SCD** | 0.841 | 6.38E-03 | AMPK, Fatty Acid Synthesis |
| **MYBL2** | -0.344 | 6.38E-03 | Cell Cycle, Transcriptional Regulation |
| **CPT1A** | -0.739 | 6.38E-03 | AMPK, Fatty Acid Oxidation |
| **CYP1B1** | 0.371 | 8.65E-03 | TryptophanKynurenine Metabolism |
| **SREBF2** | 0.317 | 8.65E-03 | Transcriptional Regulation |
| **SLC25A1** | 0.281 | 1.11E-02 | Fatty Acid Synthesis |
| **IDH1** | 0.243 | 2.00E-02 | IDH12 Activity, Reactive Oxygen Response, Vitamin & Cofactor Metabolism |
| **TIMELESS** | -0.281 | 2.00E-02 | DNA Damage Repair |
| **ACAA2** | -0.324 | 2.00E-02 | Amino Acid Synthesis, Fatty Acid Oxidation |
| **TFRC** | -0.217 | 2.06E-02 | Endocytosis, Myc |
| **TKT** | 0.249 | 2.19E-02 | Pentose Phosphate Pathway |
| **NCOA2** | 0.183 | 3.96E-02 | Epigenetic Regulation, Mitochondrial Respiration |

| PDO-BC25 TVB-2640 vs. DMSO | | | |
|---|---|---|---|
| **Gene** | **Log2 Fold Change** | **p* value** | **Gene Set** |
| **SCD** | 0.332 | 3.82E-04 | AMPK, Fatty Acid Synthesis |
| **KMT2E** | -0.232 | 3.07E-03 | Amino Acid Synthesis, Epigenetic Regulation, Transcriptional Regulation |
| **FASN** | 0.316 | 4.16E-02 | AMPK, Fatty Acid Synthesis, Myc, Vitamin & Cofactor Metabolism |
| **ATP6V1F** | 0.109 | 4.16E-02 | Mitochondrial Respiration, mTOR |
| **RRM1** | -0.277 | 4.16E-02 | Nucleotide Synthesis |
| **THBS2** | -0.332 | 4.16E-02 | PI3K |

| PDO-BC25-2 TVB-2640 vs. DMSO | | | |
|---|---|---|---|
| **Gene** | **Log2 Fold Change** | **p* value** | **Gene Set** |
| **SCD** | 0.522 | 2.45E-02 | AMPK, Fatty Acid Synthesis |
| **SREBF1** | 0.322 | 2.45E-02 | AMPK, Transcriptional Regulation |
| **FNIP2** | 0.309 | 2.45E-02 | mTOR |
| **NEU1** | 0.203 | 2.45E-02 | Lysosomal Degradation |
| **GBA** | 0.202 | 2.45E-02 | Lysosomal Degradation |
| **HEXB** | 0.171 | 2.45E-02 | Lysosomal Degradation |
| **MGST3** | 0.0882 | 2.45E-02 | KEAP1NRF2 Pathway |
| **FANCD2** | -0.504 | 2.45E-02 | DNA Damage Repair |
| **HMOX1** | 0.254 | 2.49E-02 | Cytokine & Chemokine Signaling, KEAP1NRF2 Pathway, Reactive Oxygen Response |
| **THBS1** | -0.33 | 2.49E-02 | Myc, PI3K |
| **CCNA2** | -0.339 | 2.49E-02 | AMPK, Cell Cycle, DNA Damage Repair, Myc |
| **MKI67** | -0.746 | 2.49E-02 | Cell Cycle |
| **PSAT1** | 0.197 | 3.12E-02 | Amino Acid Synthesis, Glutamine Metabolism |
| **GLRX** | 0.172 | 3.34E-02 | Glutamine Metabolism |
| **HLA-A** | 0.176 | 3.51E-02 | Antigen Presentation, Cytokine & Chemokine Signaling, Endocytosis |
| **CCL5** | 0.865 | 3.61E-02 | Cytokine & Chemokine Signaling |
| **SEC13** | 0.205 | 3.83E-02 | Antigen Presentation, Cell Cycle, Glycolysis, mTOR |
| **TXN** | 0.0917 | 4.75E-02 | p53 Pathway |
| **TALDO1** | 0.123 | 4.89E-02 | Cytokine & Chemokine Signaling, Pentose Phosphate Pathway |
| **RRM2** | -0.31 | 4.93E-02 | Cell Cycle, Nucleotide Synthesis |
| **MYBL2** | -0.441 | 4.93E-02 | Cell Cycle, Transcriptional Regulation |

| Combined DE Analysis TVB-3166 vs. DMSO | | | |
|---|---|---|---|
| **Gene** | **Log2 Fold Change** | **p* value** | **Gene Set** |
| **SLC7A11** | 0.338 | 7.93E-05 | Amino Acid Transporters, Reactive Oxygen Response |
| **CDC20** | -0.265 | 9.15E-05 | Antigen Presentation, Cell Cycle |
| **SREBF1** | 0.237 | 4.68E-04 | AMPK, Transcriptional Regulation |
| **TPX2** | -0.37 | 6.35E-04 | Cell Cycle |
| **AOX1** | -0.477 | 1.05E-03 | Amino Acid Synthesis, Cytokine & Chemokine Signaling, TryptophanKynurenine Metabolism, Vitamin & Cofactor Metabolism |
| **ATP6V1F** | 0.127 | 1.62E-03 | Mitochondrial Respiration, mTOR |
| **NQO1** | 0.342 | 2.02E-03 | KEAP1NRF2 Pathway, Reactive Oxygen Response |
| **RRM2** | -0.325 | 2.21E-03 | Cell Cycle, Nucleotide Synthesis |
| **CAT** | -0.163 | 5.94E-03 | Reactive Oxygen Response, TryptophanKynurenine Metabolism |
| **SEC13** | 0.14 | 6.86E-03 | Antigen Presentation, Cell Cycle, Glycolysis, mTOR |
| **PTGES** | 0.552 | 8.70E-03 | Glutamine Metabolism |
| **TYMS** | -0.291 | 9.18E-03 | Cell Cycle, Nucleotide Synthesis |
| **PDK4** | -0.806 | 9.18E-03 | Mitochondrial Respiration |
| **IDH2** | -0.213 | 9.22E-03 | IDH12 Activity, Mitochondrial Respiration |
| **FASN** | 0.305 | 1.21E-02 | AMPK, Fatty Acid Synthesis, Myc, Vitamin & Cofactor Metabolism |
| **CTSL** | 0.156 | 1.75E-02 | Antigen Presentation, Autophagy, Lysosomal Degradation, TLR Signaling |
| **RRM1** | -0.181 | 1.75E-02 | Nucleotide Synthesis |
| **CYP1B1** | 0.419 | 1.76E-02 | TryptophanKynurenine Metabolism |
| **UPP1** | 0.33 | 1.76E-02 | Nucleotide Salvage, Nucleotide Synthesis |
| **HMOX1** | 0.312 | 1.76E-02 | Cytokine & Chemokine Signaling, KEAP1NRF2 Pathway, Reactive Oxygen Response |
| **SLC3A2** | 0.297 | 1.76E-02 | Amino Acid Transporters, mTOR |
| **SQSTM1** | 0.237 | 1.76E-02 | Cytokine & Chemokine Signaling |
| **EGFR** | 0.162 | 1.76E-02 | Cytokine & Chemokine Signaling, Endocytosis, MAPK, PI3K, Reactive Oxygen Response |

| | | | |
|---|---|---|---|
| **KPNA2** | -0.137 | 1.76E-02 | Cytokine & Chemokine Signaling, DNA Damage Repair |
| **ZNF85** | -0.346 | 1.76E-02 | Transcriptional Regulation |
| **GTSE1** | -0.465 | 1.76E-02 | Cell Cycle |
| **CA9** | -0.525 | 1.76E-02 | Hypoxia |
| **UBE2C** | -0.195 | 2.06E-02 | Antigen Presentation, Cell Cycle |
| **ACACA** | 0.383 | 2.81E-02 | AMPK, Fatty Acid Synthesis, Vitamin & Cofactor Metabolism |
| **PGM2** | -0.203 | 2.81E-02 | Glycolysis, Nucleotide Synthesis, Pentose Phosphate Pathway |
| **BRIP1** | -0.266 | 2.81E-02 | Cell Cycle, DNA Damage Repair |
| **CENPA** | -0.24 | 3.45E-02 | Cell Cycle |
| **KYNU** | 0.239 | 3.63E-02 | TryptophanKynurenine Metabolism |
| **SHMT2** | 0.108 | 3.83E-02 | Amino Acid Synthesis, Vitamin & Cofactor Metabolism |
| **HADH** | -0.121 | 3.83E-02 | Amino Acid Synthesis, Fatty Acid Oxidation, TryptophanKynurenine Metabolism |
| **DTL** | -0.77 | 3.83E-02 | DNA Damage Repair |
| **RAD51** | -0.931 | 3.83E-02 | Cell Cycle, DNA Damage Repair |
| **PRIM2** | -0.195 | 3.89E-02 | Cell Cycle, Nucleotide Synthesis |
| **BUB1** | -0.218 | 3.89E-02 | Cell Cycle |
| **TK1** | -0.506 | 3.89E-02 | Cell Cycle, Nucleotide Salvage, Nucleotide Synthesis |
| **SLC1A5** | 0.261 | 4.00E-02 | Amino Acid Transporters |
| **CCNB2** | -0.246 | 4.18E-02 | Cell Cycle |

| MDA-MB-231-BR TVB-3166 vs. DMSO | | | |
|---|---|---|---|
| **Gene** | **Log2 Fold Change** | **p* value** | **Gene Set** |
| **SCD** | 0.558 | 5.35E-08 | AMPK, Fatty Acid Synthesis |
| **SREBF1** | 0.466 | 2.99E-05 | AMPK, Transcriptional Regulation |
| **SLC7A11** | 0.622 | 1.14E-04 | Amino Acid Transporters, Reactive Oxygen Response |
| **SLC3A2** | 0.718 | 1.48E-04 | Amino Acid Transporters, mTOR |
| **PTGS2** | 1.24 | 1.70E-04 | Cytokine & Chemokine Signaling, NF-kB, Reactive Oxygen Response, Vitamin & Cofactor Metabolism |
| **NQO1** | 0.936 | 1.70E-04 | KEAP1NRF2 Pathway, Reactive Oxygen Response |
| **UPP1** | 0.667 | 1.70E-04 | Nucleotide Salvage, Nucleotide Synthesis |
| **KYNU** | 0.633 | 1.70E-04 | TryptophanKynurenine Metabolism |
| **CMKLR1** | 0.562 | 1.70E-04 | MAPK, PI3K |
| **CA9** | -1.26 | 1.70E-04 | Hypoxia |
| **FASN** | 0.62 | 2.22E-04 | AMPK, Fatty Acid Synthesis, Myc, Vitamin & Cofactor Metabolism |
| **PTGES** | 0.948 | 2.28E-04 | Glutamine Metabolism |
| **NPM1** | -0.254 | 2.28E-04 | Cell Cycle, Myc |
| **LAMA4** | -0.702 | 3.91E-04 | PI3K |
| **IMPDH2** | -0.25 | 4.48E-04 | Nucleotide Synthesis |
| **LAMB1** | -0.52 | 4.60E-04 | PI3K |
| **TXNRD1** | 0.395 | 4.80E-04 | p53 Pathway |
| **SOD2** | 0.335 | 4.91E-04 | Cytokine & Chemokine Signaling, Mitochondrial Respiration |
| **GAPDH** | -0.336 | 5.11E-04 | Glycolysis |
| **RRM2** | -0.347 | 6.75E-04 | Cell Cycle, Nucleotide Synthesis |
| **CYP1B1** | 1.06 | 7.70E-04 | TryptophanKynurenine Metabolism |
| **PGK1** | -0.536 | 1.07E-03 | Glycolysis |
| **RAD51AP1** | -0.533 | 1.12E-03 | DNA Damage Repair |

| | | | |
|---|---|---|---|
| **SQSTM1** | 0.439 | 1.17E-03 | Cytokine & Chemokine Signaling |
| **PEBP1** | -0.237 | 1.21E-03 | Reactive Oxygen Response |
| **TLR4** | 0.379 | 1.28E-03 | Antigen Presentation, NF-kB, PI3K, TLR Signaling |
| **CAT** | -0.373 | 1.28E-03 | Reactive Oxygen Response, TryptophanKynurenine Metabolism |
| **PRIM1** | -0.425 | 1.47E-03 | Cell Cycle, Nucleotide Synthesis |
| **LTA4H** | -0.264 | 1.63E-03 | Amino Acid Synthesis |
| **PGM2** | -0.356 | 1.63E-03 | Glycolysis, Nucleotide Synthesis, Pentose Phosphate Pathway |
| **CA12** | -0.791 | 1.63E-03 | Reactive Oxygen Response |
| **NCAPH** | -0.425 | 1.96E-03 | Cell Cycle |
| **SLC7A5** | 0.291 | 2.90E-03 | Amino Acid Transporters, mTOR |
| **NFE2L2** | 0.151 | 3.13E-03 | IDH12 Activity, KEAP1NRF2 Pathway, Transcriptional Regulation |
| **GLUL** | -0.301 | 3.97E-03 | Amino Acid Synthesis, Arginine Metabolism, Glutamine Metabolism |
| **PDK4** | -0.818 | 3.97E-03 | Mitochondrial Respiration |
| **CTSD** | 0.583 | 4.15E-03 | Antigen Presentation, Autophagy, Lysosomal Degradation |
| **SLC1A5** | 0.516 | 4.15E-03 | Amino Acid Transporters |
| **MSH2** | -0.258 | 4.15E-03 | DNA Damage Repair |
| **RICTOR** | 0.154 | 6.40E-03 | mTOR |
| **BRIP1** | -0.423 | 6.48E-03 | Cell Cycle, DNA Damage Repair |
| **CD63** | 0.211 | 6.49E-03 | Lysosomal Degradation |
| **RRM1** | -0.421 | 6.53E-03 | Nucleotide Synthesis |
| **GZMA** | -0.703 | 6.53E-03 | |
| **PDK1** | -0.435 | 6.72E-03 | Mitochondrial Respiration |
| **THBS1** | 0.587 | 7.18E-03 | Myc, PI3K |
| **HMOX1** | 0.679 | 7.40E-03 | Cytokine & Chemokine Signaling, KEAP1NRF2 Pathway, Reactive Oxygen Response |
| **PSAT1** | 0.223 | 7.40E-03 | Amino Acid Synthesis, Glutamine Metabolism |
| **TKT** | 0.158 | 7.40E-03 | Pentose Phosphate Pathway |
| **GUSB** | -0.23 | 7.40E-03 | Lysosomal Degradation |
| **TPR** | -0.241 | 7.40E-03 | Cell Cycle, Cytokine & Chemokine Signaling, Glycolysis |
| **LDHB** | -0.204 | 8.31E-03 | Amino Acid Synthesis, Glycolysis, Mitochondrial Respiration |
| **TYMS** | -0.269 | 8.62E-03 | Cell Cycle, Nucleotide Synthesis |
| **UBE2C** | -0.367 | 9.01E-03 | Antigen Presentation, Cell Cycle |

| | | | |
|---|---|---|---|
| **GLUD1** | -0.163 | 9.38E-03 | Amino Acid Synthesis, Arginine Metabolism, Glutamine Metabolism, Mitochondrial Respiration |
| **HPRT1** | -0.233 | 9.38E-03 | Nucleotide Salvage, Nucleotide Synthesis |
| **RAD51** | -0.44 | 9.38E-03 | Cell Cycle, DNA Damage Repair |
| **HACD2** | 0.311 | 9.70E-03 | Fatty Acid Synthesis |
| **ACAA2** | -0.247 | 9.94E-03 | Amino Acid Synthesis, Fatty Acid Oxidation |
| **HADH** | -0.263 | 1.07E-02 | Amino Acid Synthesis, Fatty Acid Oxidation, TryptophanKynurenine Metabolism |
| **VEGFA** | 0.333 | 1.18E-02 | Cytokine & Chemokine Signaling, Hypoxia, MAPK, PI3K |
| **ACAT1** | -0.272 | 1.21E-02 | Amino Acid Synthesis, Fatty Acid Oxidation, TryptophanKynurenine Metabolism |
| **AOX1** | -0.512 | 1.21E-02 | Amino Acid Synthesis, Cytokine & Chemokine Signaling, TryptophanKynurenine Metabolism, Vitamin & Cofactor Metabolism |
| **GPI** | -0.463 | 1.24E-02 | Glycolysis, p53 Pathway, Pentose Phosphate Pathway |
| **ENO1** | -0.293 | 1.29E-02 | Glycolysis, Myc |
| **KIF2C** | -0.304 | 1.32E-02 | Antigen Presentation, Cell Cycle |
| **ALDOA** | -0.206 | 1.35E-02 | Glycolysis, Pentose Phosphate Pathway |
| **MAP1LC3B** | 0.163 | 1.42E-02 | Autophagy |
| **PFKL** | -0.243 | 1.60E-02 | AMPK, Glycolysis, Pentose Phosphate Pathway |
| **SOD3** | -0.306 | 1.63E-02 | Reactive Oxygen Response |
| **HK2** | -0.282 | 1.69E-02 | Glycolysis |
| **DCK** | -0.162 | 1.76E-02 | Nucleotide Salvage, Nucleotide Synthesis |
| **TK1** | -0.268 | 1.76E-02 | Cell Cycle, Nucleotide Salvage, Nucleotide Synthesis |
| **ERN1** | 0.396 | 2.06E-02 | Autophagy |
| **ATF4** | 0.19 | 2.12E-02 | MAPK, PI3K, Transcriptional Regulation |
| **IL6** | 0.492 | 2.13E-02 | Cytokine & Chemokine Signaling, PI3K |
| **SMAD3** | 0.178 | 2.13E-02 | Cell Cycle, Cytokine & Chemokine Signaling, Endocytosis |
| **EHHADH** | -0.426 | 2.32E-02 | Amino Acid Synthesis, Fatty Acid Oxidation, TryptophanKynurenine Metabolism |
| **SHMT2** | 0.209 | 2.35E-02 | Amino Acid Synthesis, Vitamin & Cofactor Metabolism |
| **IDH2** | -0.458 | 2.35E-02 | IDH12 Activity, Mitochondrial Respiration |
| **BUB1B** | -0.23 | 2.44E-02 | Cell Cycle |
| **TP53** | -0.238 | 2.52E-02 | Cell Cycle, Cytokine & Chemokine Signaling, DNA Damage Repair, MAPK, Myc, p53 Pathway, PI3K, Transcriptional Regulation |
| **CPS1** | -0.663 | 2.79E-02 | Amino Acid Synthesis, Arginine Metabolism |
| **CBR4** | -0.231 | 2.82E-02 | Fatty Acid Synthesis |
| **GTSE1** | -0.253 | 2.86E-02 | Cell Cycle |

| | | | |
|---|---|---|---|
| **PDK3** | -0.282 | 2.90E-02 | Mitochondrial Respiration |
| **TK2** | 0.214 | 2.97E-02 | Nucleotide Salvage, Nucleotide Synthesis |
| **SOS2** | 0.223 | 3.07E-02 | Cytokine & Chemokine Signaling, MAPK, mTOR, PI3K, TCR & Costimulatory Signaling |
| **DTL** | -0.438 | 3.16E-02 | DNA Damage Repair |
| **KYAT3** | -0.196 | 3.31E-02 | Amino Acid Synthesis, TryptophanKynurenine Metabolism |
| **NT5E** | -0.166 | 3.35E-02 | Nucleotide Synthesis, Vitamin & Cofactor Metabolism |
| **HEXA** | -0.182 | 3.46E-02 | Lysosomal Degradation |
| **CD274** | -0.849 | 3.73E-02 | TCR & Costimulatory Signaling |
| **GMPR2** | -0.148 | 3.81E-02 | Nucleotide Salvage, Nucleotide Synthesis |
| **BUB1** | -0.29 | 3.81E-02 | Cell Cycle |
| **TBK1** | 0.119 | 4.14E-02 | Cytokine & Chemokine Signaling, TLR Signaling |
| **CYP1A1** | 1.21 | 4.20E-02 | TryptophanKynurenine Metabolism |
| **AFMID** | -0.342 | 4.20E-02 | TryptophanKynurenine Metabolism |
| **PRKAG2** | 0.175 | 4.28E-02 | AMPK, Autophagy, Fatty Acid Oxidation, Mitochondrial Respiration, mTOR, p53 Pathway |
| **GMPR** | 0.294 | 4.50E-02 | Nucleotide Salvage, Nucleotide Synthesis |
| **CCNB2** | -0.185 | 4.53E-02 | Cell Cycle |
| **CDC20** | -0.245 | 4.53E-02 | Antigen Presentation, Cell Cycle |
| **PRPS1** | -0.237 | 4.67E-02 | Nucleotide Synthesis, Pentose Phosphate Pathway |
| **TPX2** | -0.272 | 4.67E-02 | Cell Cycle |
| **ZNF675** | -0.471 | 4.67E-02 | Transcriptional Regulation |
| **ITGB1** | -0.0916 | 4.77E-02 | Cytokine & Chemokine Signaling, PI3K |
| **NDUFB4** | -0.0956 | 4.77E-02 | Mitochondrial Respiration, Reactive Oxygen Response |
| **NME2** | -0.107 | 4.77E-02 | Myc, Nucleotide Synthesis |
| **ACAT2** | -0.212 | 4.77E-02 | Amino Acid Synthesis, Fatty Acid Oxidation, TryptophanKynurenine Metabolism |
| **BCL2A1** | -0.293 | 4.77E-02 | NF-kB |
| **GCLC** | 0.163 | 4.78E-02 | Amino Acid Synthesis, Reactive Oxygen Response |
| **YWHAZ** | -0.114 | 4.81E-02 | Cell Cycle, Cytokine & Chemokine Signaling, p53 Pathway, PI3K |
| **H6PD** | -0.243 | 4.90E-02 | Pentose Phosphate Pathway |
| **COPS6** | -0.155 | 4.99E-02 | DNA Damage Repair, Endocytosis |

| MDA-MB-231 TVB-3166 vs. DMSO | | | |
|---|---|---|---|
| **Gene** | **Log2 Fold Change** | **p* value** | **Gene Set** |
| **FASN** | 0.791 | 9.50E-04 | AMPK, Fatty Acid Synthesis, Myc, Vitamin & Cofactor Metabolism |
| **NCOA2** | 0.433 | 9.50E-04 | Epigenetic Regulation, Mitochondrial Respiration |
| **PDK4** | -1.32 | 9.50E-04 | Mitochondrial Respiration |
| **SLC25A1** | 0.402 | 3.94E-03 | Fatty Acid Synthesis |
| **CPT1A** | -0.931 | 3.94E-03 | AMPK, Fatty Acid Oxidation |
| **CYP1B1** | 0.453 | 7.77E-03 | TryptophanKynurenine Metabolism |
| **PDP1** | -0.178 | 8.46E-03 | Mitochondrial Respiration |
| **STAT3** | 0.206 | 9.21E-03 | Cytokine & Chemokine Signaling, Transcriptional Regulation |
| **ACAT2** | 0.42 | 1.17E-02 | Amino Acid Synthesis, Fatty Acid Oxidation, TryptophanKynurenine Metabolism |
| **TFRC** | -0.28 | 1.17E-02 | Endocytosis, Myc |
| **GLUL** | 0.177 | 1.42E-02 | Amino Acid Synthesis, Arginine Metabolism, Glutamine Metabolism |
| **PTGS2** | -0.48 | 1.42E-02 | Cytokine & Chemokine Signaling, NF-kB, Reactive Oxygen Response, Vitamin & Cofactor Metabolism |
| **NQO1** | 0.331 | 1.55E-02 | KEAP1NRF2 Pathway, Reactive Oxygen Response |
| **TYMS** | -0.219 | 1.55E-02 | Cell Cycle, Nucleotide Synthesis |
| **MAP2K3** | 0.237 | 1.60E-02 | Cytokine & Chemokine Signaling, MAPK, TLR Signaling |
| **IDH1** | 0.275 | 1.79E-02 | IDH12 Activity, Reactive Oxygen Response, Vitamin & Cofactor Metabolism |
| **TALDO1** | 0.136 | 2.19E-02 | Cytokine & Chemokine Signaling, Pentose Phosphate Pathway |
| **SREBF1** | 0.33 | 2.64E-02 | AMPK, Transcriptional Regulation |
| **MYBL1** | -0.473 | 2.64E-02 | Transcriptional Regulation |
| **SREBF2** | 0.276 | 3.14E-02 | Transcriptional Regulation |
| **PIK3CD** | 0.264 | 3.18E-02 | AMPK, Autophagy, Cytokine & Chemokine Signaling, mTOR, PI3K, TCR & Costimulatory Signaling |
| **CD276** | 0.216 | 3.40E-02 | TCR & Costimulatory Signaling |
| **GBA** | 0.218 | 3.83E-02 | Lysosomal Degradation |

| | | | |
|---|---|---|---|
| **GLUD1** | 0.114 | 3.83E-02 | Amino Acid Synthesis, Arginine Metabolism, Glutamine Metabolism, Mitochondrial Respiration |
| **SCD** | 0.615 | 4.11E-02 | AMPK, Fatty Acid Synthesis |
| **HEXA** | 0.208 | 4.11E-02 | Lysosomal Degradation |
| **CD63** | 0.188 | 4.11E-02 | Lysosomal Degradation |
| **HIF1A** | 0.17 | 4.11E-02 | Autophagy, Cytokine & Chemokine Signaling, Hypoxia, Transcriptional Regulation |
| **SLC16A3** | 0.158 | 4.11E-02 | Mitochondrial Respiration |
| **SMAD2** | 0.115 | 4.11E-02 | Cell Cycle, Endocytosis |
| **COX8A** | 0.11 | 4.11E-02 | Mitochondrial Respiration, p53 Pathway |
| **ACAA2** | -0.292 | 4.11E-02 | Amino Acid Synthesis, Fatty Acid Oxidation |
| **CDCA5** | -0.308 | 4.11E-02 | Cell Cycle |
| **AOX1** | -0.591 | 4.54E-02 | Amino Acid Synthesis, Cytokine & Chemokine Signaling, TryptophanKynurenine Metabolism, Vitamin & Cofactor Metabolism |

| PDO-BC25 TVB-3166 vs. DMSO | | | |
|---|---|---|---|
| **Gene** | **Log2 Fold Change** | **p* value** | **Gene Set** |
| **PRKAG1** | 0.207 | 2.12E-02 | AMPK, Autophagy, Mitochondrial Respiration, mTOR, p53 Pathway |
| **CTSS** | 0.236 | 2.43E-02 | Antigen Presentation, Lysosomal Degradation, TLR Signaling |
| **MLST8** | 0.365 | 4.41E-02 | Autophagy, mTOR, p53 Pathway, PI3K |
| **THBS2** | -0.328 | 4.62E-02 | PI3K |
| **UPP1** | 0.257 | 4.62E-02 | Nucleotide Salvage, Nucleotide Synthesis |
| **EFNA4** | -0.32 | 4.84E-02 | MAPK, PI3K |

| PDO-BC25-2 TVB-3166 vs. DMSO | | | |
|---|---|---|---|
| **Gene** | **Log2 Fold Change** | **p* value** | **Gene Set** |
| **HMOX1** | 0.522 | 2.09E-03 | Cytokine & Chemokine Signaling, KEAP1NRF2 Pathway, Reactive Oxygen Response |
| **PSAT1** | 0.454 | 2.09E-03 | Amino Acid Synthesis, Glutamine Metabolism |
| **FNIP2** | 0.487 | 2.69E-03 | mTOR |
| **GLRX** | 0.339 | 2.69E-03 | Glutamine Metabolism |
| **HLA-C** | 0.264 | 2.69E-03 | Antigen Presentation, Cytokine & Chemokine Signaling, Endocytosis |
| **RRM2** | -0.643 | 3.21E-03 | Cell Cycle, Nucleotide Synthesis |
| **THBS1** | -0.537 | 4.62E-03 | Myc, PI3K |
| **CCNA2** | -0.522 | 5.29E-03 | AMPK, Cell Cycle, DNA Damage Repair, Myc |
| **STAM2** | 0.322 | 6.88E-03 | Cytokine & Chemokine Signaling, Endocytosis |
| **GBA** | 0.278 | 6.88E-03 | Lysosomal Degradation |
| **PLK1** | -0.785 | 6.88E-03 | Cell Cycle |
| **SEC13** | 0.332 | 6.91E-03 | Antigen Presentation, Cell Cycle, Glycolysis, mTOR |
| **CENPA** | -0.674 | 8.84E-03 | Cell Cycle |
| **MKI67** | -1.02 | 9.16E-03 | Cell Cycle |
| **SQSTM1** | 0.44 | 1.00E-02 | Cytokine & Chemokine Signaling |
| **CCNB2** | -0.463 | 1.03E-02 | Cell Cycle |
| **CMKLR1** | -0.496 | 1.03E-02 | MAPK, PI3K |
| **LY96** | 0.291 | 1.14E-02 | Antigen Presentation, NF-kB, TLR Signaling |
| **CHMP2A** | 0.214 | 1.26E-02 | Autophagy, Endocytosis |
| **KPNA2** | -0.286 | 1.26E-02 | Cytokine & Chemokine Signaling, DNA Damage Repair |
| **ATP6V1F** | 0.248 | 1.30E-02 | Mitochondrial Respiration, mTOR |
| **MYBL2** | -0.643 | 1.37E-02 | Cell Cycle, Transcriptional Regulation |
| **ITGA11** | -0.263 | 1.47E-02 | PI3K |

| | | | |
|---|---|---|---|
| CDCA8 | -0.828 | 1.47E-02 | Cell Cycle |
| VEGFA | 0.243 | 1.83E-02 | Cytokine & Chemokine Signaling, Hypoxia, MAPK, PI3K |
| CYP1B1 | 0.643 | 1.84E-02 | TryptophanKynurenine Metabolism |
| CTSL | 0.317 | 1.84E-02 | Antigen Presentation, Autophagy, Lysosomal Degradation, TLR Signaling |
| HJURP | -0.698 | 1.84E-02 | Cell Cycle |
| SOD2 | 0.631 | 1.86E-02 | Cytokine & Chemokine Signaling, Mitochondrial Respiration |
| SLC7A5 | 0.315 | 1.86E-02 | Amino Acid Transporters, mTOR |
| PRKAA1 | 0.16 | 1.86E-02 | AMPK, Autophagy, mTOR, p53 Pathway, PI3K |
| FANCD2 | -0.536 | 1.86E-02 | DNA Damage Repair |
| SLC1A5 | 0.62 | 1.95E-02 | Amino Acid Transporters |
| ATF4 | 0.347 | 1.95E-02 | MAPK, PI3K, Transcriptional Regulation |
| NFE2L2 | 0.319 | 1.95E-02 | IDH12 Activity, KEAP1NRF2 Pathway, Transcriptional Regulation |
| RAD51 | -1.83 | 1.95E-02 | Cell Cycle, DNA Damage Repair |
| LAMTOR5 | 0.166 | 2.06E-02 | Autophagy, mTOR, p53 Pathway |
| HK2 | 0.364 | 2.23E-02 | Glycolysis |
| SLC25A1 | 0.282 | 2.25E-02 | Fatty Acid Synthesis |
| RUNX2 | -0.497 | 2.25E-02 | Transcriptional Regulation |
| UBE2T | -0.421 | 2.30E-02 | DNA Damage Repair |
| PHGDH | 0.311 | 2.47E-02 | Amino Acid Synthesis, Glutamine Metabolism |
| CDC20 | -0.343 | 2.47E-02 | Antigen Presentation, Cell Cycle |
| SLC3A2 | 0.431 | 2.62E-02 | Amino Acid Transporters, mTOR |
| NEU1 | 0.174 | 2.62E-02 | Lysosomal Degradation |
| RUNX1 | -0.352 | 2.62E-02 | Transcriptional Regulation |
| TFAM | 0.402 | 2.76E-02 | Mitochondrial Respiration |
| MAT2A | -0.34 | 2.84E-02 | Amino Acid Synthesis |
| HLA-A | 0.184 | 3.12E-02 | Antigen Presentation, Cytokine & Chemokine Signaling, Endocytosis |
| RRAGC | 0.261 | 3.29E-02 | Autophagy, mTOR, p53 Pathway |
| CD68 | 0.447 | 3.32E-02 | Lysosomal Degradation |
| MYC | 0.349 | 3.32E-02 | Cell Cycle, Cytokine & Chemokine Signaling, MAPK, Myc, PI3K, Transcriptional Regulation |
| PYCR1 | 0.335 | 3.32E-02 | Amino Acid Synthesis, Glutamine Metabolism |
| HEXA | 0.161 | 3.32E-02 | Lysosomal Degradation |

| | | | |
|---|---|---|---|
| **TYMS** | -0.7 | 3.32E-02 | Cell Cycle, Nucleotide Synthesis |
| **GTSE1** | -0.727 | 3.32E-02 | Cell Cycle |
| **COX6A1** | 0.131 | 3.33E-02 | Mitochondrial Respiration, p53 Pathway |
| **CCL5** | 0.874 | 3.35E-02 | Cytokine & Chemokine Signaling |
| **PTGES** | 0.527 | 3.35E-02 | Glutamine Metabolism |
| **EGFR** | 0.255 | 3.35E-02 | Cytokine & Chemokine Signaling, Endocytosis, MAPK, PI3K, Reactive Oxygen Response |
| **CA9** | 0.21 | 3.35E-02 | Hypoxia |
| **HEXB** | 0.139 | 3.35E-02 | Lysosomal Degradation |
| **ASNS** | 0.346 | 3.44E-02 | Amino Acid Synthesis, Glutamine Metabolism |
| **NPM1** | 0.137 | 3.44E-02 | Cell Cycle, Myc |
| **PGK1** | -0.216 | 3.47E-02 | Glycolysis |
| **BUB1B** | -0.464 | 3.60E-02 | Cell Cycle |
| **HK1** | 0.274 | 3.66E-02 | Glycolysis |
| **XRCC2** | -1.46 | 3.66E-02 | DNA Damage Repair |
| **ERN1** | 0.416 | 3.83E-02 | Autophagy |
| **GAPDH** | 0.174 | 3.83E-02 | Glycolysis |
| **COX4I1** | 0.16 | 3.89E-02 | Mitochondrial Respiration, p53 Pathway |
| **MCAT** | 1.22 | 3.97E-02 | Fatty Acid Oxidation, Fatty Acid Synthesis |
| **COX5A** | 0.0736 | 4.15E-02 | Mitochondrial Respiration, p53 Pathway |
| **PDCD1LG2** | 0.785 | 4.24E-02 | TCR & Costimulatory Signaling |
| **TALDO1** | 0.121 | 4.68E-02 | Cytokine & Chemokine Signaling, Pentose Phosphate Pathway |
| **SMAD3** | -0.262 | 4.85E-02 | Cell Cycle, Cytokine & Chemokine Signaling, Endocytosis |

**Combined DE Analysis BI-99179 vs. DMSO**

| Gene | Log2 Fold Change | p* value | Gene Set |
|---|---|---|---|
| **SLC7A11** | 0.431 | 1.77E-07 | Amino Acid Transporters, Reactive Oxygen Response |
| **FASN** | 0.43 | 4.24E-04 | AMPK, Fatty Acid Synthesis, Myc, Vitamin & Cofactor Metabolism |
| **SREBF1** | 0.238 | 4.24E-04 | AMPK, Transcriptional Regulation |
| **SCD** | 0.591 | 8.71E-04 | AMPK, Fatty Acid Synthesis |
| **CDC20** | -0.208 | 2.79E-03 | Antigen Presentation, Cell Cycle |
| **AOX1** | -0.444 | 2.79E-03 | Amino Acid Synthesis, Cytokine & Chemokine Signaling, TryptophanKynurenine Metabolism, Vitamin & Cofactor Metabolism |
| **PGM2** | -0.275 | 2.91E-03 | Glycolysis, Nucleotide Synthesis, Pentose Phosphate Pathway |
| **ATP6V1F** | 0.111 | 9.20E-03 | Mitochondrial Respiration, mTOR |
| **NQO1** | 0.297 | 1.10E-02 | KEAP1NRF2 Pathway, Reactive Oxygen Response |
| **CA9** | -0.579 | 1.10E-02 | Hypoxia |
| **ACACA** | 0.441 | 1.74E-02 | AMPK, Fatty Acid Synthesis, Vitamin & Cofactor Metabolism |
| **TK2** | 0.159 | 3.10E-02 | Nucleotide Salvage, Nucleotide Synthesis |
| **IDH2** | -0.188 | 4.24E-02 | IDH12 Activity, Mitochondrial Respiration |
| **PTGES** | 0.472 | 4.25E-02 | Glutamine Metabolism |

**MDA-MB-231-BR BI-99179 vs. DMSO**

| Gene | Log2 Fold Change | p* value | Gene Set |
|---|---|---|---|
| SCD | 0.762 | 1.81E-09 | AMPK, Fatty Acid Synthesis |
| SREBF1 | 0.555 | 4.86E-06 | AMPK, Transcriptional Regulation |
| SLC7A11 | 0.674 | 5.01E-05 | Amino Acid Transporters, Reactive Oxygen Response |
| PTGS2 | 1.34 | 1.30E-04 | Cytokine & Chemokine Signaling, NF-kB, Reactive Oxygen Response, Vitamin & Cofactor Metabolism |
| UPP1 | 0.719 | 1.36E-04 | Nucleotide Salvage, Nucleotide Synthesis |
| SLC3A2 | 0.688 | 1.36E-04 | Amino Acid Transporters, mTOR |
| FASN | 0.68 | 1.36E-04 | AMPK, Fatty Acid Synthesis, Myc, Vitamin & Cofactor Metabolism |
| KYNU | 0.64 | 1.36E-04 | TryptophanKynurenine Metabolism |
| SOD2 | 0.401 | 1.80E-04 | Cytokine & Chemokine Signaling, Mitochondrial Respiration |
| CA9 | -1.12 | 3.41E-04 | Hypoxia |
| TXNRD1 | 0.394 | 7.45E-04 | p53 Pathway |
| PTGES | 0.843 | 7.45E-04 | Glutamine Metabolism |
| TLR4 | 0.433 | 8.15E-04 | Antigen Presentation, NF-kB, PI3K, TLR Signaling |
| CD63 | 0.306 | 8.20E-04 | Lysosomal Degradation |
| RICTOR | 0.216 | 9.75E-04 | mTOR |
| NQO1 | 0.738 | 9.89E-04 | KEAP1NRF2 Pathway, Reactive Oxygen Response |
| PSAT1 | 0.31 | 1.28E-03 | Amino Acid Synthesis, Glutamine Metabolism |
| GAPDH | -0.303 | 1.28E-03 | Glycolysis |
| SQSTM1 | 0.447 | 1.28E-03 | Cytokine & Chemokine Signaling |
| LAMA4 | -0.58 | 1.50E-03 | PI3K |
| CYP1B1 | 0.981 | 1.50E-03 | TryptophanKynurenine Metabolism |
| LAMB1 | -0.44 | 1.50E-03 | PI3K |
| FBP1 | -1.76 | 1.61E-03 | AMPK, Glycolysis, Pentose Phosphate Pathway |

| | | | |
|---|---|---|---|
| RRM2 | -0.308 | 1.61E-03 | Cell Cycle, Nucleotide Synthesis |
| NFE2L2 | 0.169 | 1.65E-03 | IDH12 Activity, KEAP1NRF2 Pathway, Transcriptional Regulation |
| CMKLR1 | 0.392 | 1.73E-03 | MAPK, PI3K |
| PDK4 | -0.921 | 1.96E-03 | Mitochondrial Respiration |
| NPM1 | -0.182 | 2.00E-03 | Cell Cycle, Myc |
| NCAPH | -0.427 | 2.04E-03 | Cell Cycle |
| MAP1LC3B | 0.233 | 2.04E-03 | Autophagy |
| SLC7A5 | 0.297 | 2.59E-03 | Amino Acid Transporters, mTOR |
| LTA4H | -0.235 | 4.06E-03 | Amino Acid Synthesis |
| IMPDH2 | -0.177 | 4.08E-03 | Nucleotide Synthesis |
| TK1 | -0.362 | 4.08E-03 | Cell Cycle, Nucleotide Salvage, Nucleotide Synthesis |
| RAD51AP1 | -0.422 | 5.15E-03 | DNA Damage Repair |
| PGK1 | -0.419 | 5.15E-03 | Glycolysis |
| PPM1A | 0.156 | 5.24E-03 | MAPK, mTOR |
| BUB1 | -0.439 | 5.41E-03 | Cell Cycle |
| ATF4 | 0.249 | 5.76E-03 | MAPK, PI3K, Transcriptional Regulation |
| PRIM1 | -0.345 | 5.76E-03 | Cell Cycle, Nucleotide Synthesis |
| PGM2 | -0.293 | 5.80E-03 | Glycolysis, Nucleotide Synthesis, Pentose Phosphate Pathway |
| RRM1 | -0.43 | 5.80E-03 | Nucleotide Synthesis |
| ACAA2 | -0.277 | 5.80E-03 | Amino Acid Synthesis, Fatty Acid Oxidation |
| BUB1B | -0.306 | 5.95E-03 | Cell Cycle |
| SLC1A5 | 0.478 | 6.53E-03 | Amino Acid Transporters |
| ASNS | 0.515 | 8.62E-03 | Amino Acid Synthesis, Glutamine Metabolism |
| VEGFA | 0.35 | 1.06E-02 | Cytokine & Chemokine Signaling, Hypoxia, MAPK, PI3K |
| ENO1 | -0.314 | 1.06E-02 | Glycolysis, Myc |
| UBE2C | -0.361 | 1.10E-02 | Antigen Presentation, Cell Cycle |
| GTSE1 | -0.315 | 1.10E-02 | Cell Cycle |
| DTL | -0.55 | 1.21E-02 | DNA Damage Repair |
| PEBP1 | -0.162 | 1.24E-02 | Reactive Oxygen Response |
| TBK1 | 0.156 | 1.25E-02 | Cytokine & Chemokine Signaling, TLR Signaling |
| HACD2 | 0.303 | 1.25E-02 | Fatty Acid Synthesis |
| ASL | -0.362 | 1.25E-02 | Amino Acid Synthesis, Arginine Metabolism, IDH12 Activity |
| IDH3A | 0.184 | 1.25E-02 | Mitochondrial Respiration |
| SOS2 | 0.272 | 1.25E-02 | Cytokine & Chemokine Signaling, MAPK, mTOR, PI3K, TCR & Costimulatory Signaling |
| SHMT2 | 0.24 | 1.26E-02 | Amino Acid Synthesis, Vitamin & Cofactor Metabolism |
| ATG101 | 0.228 | 1.33E-02 | Autophagy |
| CD274 | -1.06 | 1.38E-02 | TCR & Costimulatory Signaling |
| CA12 | -0.551 | 1.38E-02 | Reactive Oxygen Response |

| | | | |
|---|---|---|---|
| **CAT** | -0.248 | 1.38E-02 | Reactive Oxygen Response, TryptophanKynurenine Metabolism |
| **HLA-C** | 0.326 | 1.43E-02 | Antigen Presentation, Cytokine & Chemokine Signaling, Endocytosis |
| **BRIP1** | -0.355 | 1.51E-02 | Cell Cycle, DNA Damage Repair |
| **HK2** | -0.287 | 1.62E-02 | Glycolysis |
| **KIF2C** | -0.295 | 1.62E-02 | Antigen Presentation, Cell Cycle |
| **ALDOA** | -0.2 | 1.63E-02 | Glycolysis, Pentose Phosphate Pathway |
| **ECHS1** | -0.137 | 1.72E-02 | Amino Acid Synthesis, Fatty Acid Oxidation, TryptophanKynurenine Metabolism |
| **TPR** | -0.205 | 1.74E-02 | Cell Cycle, Cytokine & Chemokine Signaling, Glycolysis |
| **CTSD** | 0.443 | 1.74E-02 | Antigen Presentation, Autophagy, Lysosomal Degradation |
| **SOD3** | -0.302 | 1.74E-02 | Reactive Oxygen Response |
| **PTK2** | 0.146 | 1.78E-02 | Cytokine & Chemokine Signaling, PI3K |
| **PDK1** | -0.355 | 1.80E-02 | Mitochondrial Respiration |
| **FNIP1** | 0.238 | 1.87E-02 | mTOR |
| **ERN1** | 0.401 | 1.87E-02 | Autophagy |
| **PRKAG2** | 0.208 | 1.91E-02 | AMPK, Autophagy, Fatty Acid Oxidation, Mitochondrial Respiration, mTOR, p53 Pathway |
| **TLR10** | 2.26 | 1.91E-02 | TLR Signaling |
| **IL6** | 0.492 | 2.07E-02 | Cytokine & Chemokine Signaling, PI3K |
| **PSMC1** | 0.178 | 2.07E-02 | Antigen Presentation, Cell Cycle, Cytokine & Chemokine Signaling, Hypoxia |
| **AOX1** | -0.448 | 2.32E-02 | Amino Acid Synthesis, Cytokine & Chemokine Signaling, TryptophanKynurenine Metabolism, Vitamin & Cofactor Metabolism |
| **TP53** | -0.241 | 2.32E-02 | Cell Cycle, Cytokine & Chemokine Signaling, DNA Damage Repair, MAPK, Myc, p53 Pathway, PI3K, Transcriptional Regulation |
| **HADH** | -0.222 | 2.42E-02 | Amino Acid Synthesis, Fatty Acid Oxidation, TryptophanKynurenine Metabolism |
| **GLUL** | -0.209 | 2.43E-02 | Amino Acid Synthesis, Arginine Metabolism, Glutamine Metabolism |
| **PRKAA2** | -0.383 | 2.43E-02 | AMPK, Autophagy, Fatty Acid Oxidation, Mitochondrial Respiration, mTOR, p53 Pathway, PI3K |
| **COX14** | 0.154 | 2.43E-02 | Mitochondrial Respiration, p53 Pathway |
| **DCK** | -0.15 | 2.48E-02 | Nucleotide Salvage, Nucleotide Synthesis |
| **RB1CC1** | 0.172 | 2.52E-02 | Autophagy |
| **CLSPN** | -0.25 | 3.12E-02 | Cell Cycle, DNA Damage Repair |
| **TYMS** | -0.206 | 3.12E-02 | Cell Cycle, Nucleotide Synthesis |
| **TALDO1** | 0.155 | 3.30E-02 | Cytokine & Chemokine Signaling, Pentose Phosphate Pathway |
| **NDUFB1** | 0.112 | 3.36E-02 | Mitochondrial Respiration |
| **IDH2** | -0.422 | 3.41E-02 | IDH12 Activity, Mitochondrial Respiration |

| | | | |
|---|---|---|---|
| **CDC20** | -0.26 | 3.50E-02 | Antigen Presentation, Cell Cycle |
| **TRAF1** | 0.264 | 3.79E-02 | NF-kB |
| **LDHB** | -0.149 | 3.79E-02 | Amino Acid Synthesis, Glycolysis, Mitochondrial Respiration |
| **PDK3** | -0.262 | 3.97E-02 | Mitochondrial Respiration |
| **H6PD** | -0.259 | 3.97E-02 | Pentose Phosphate Pathway |
| **GPI** | -0.361 | 3.97E-02 | Glycolysis, p53 Pathway, Pentose Phosphate Pathway |
| **GZMA** | -0.476 | 3.99E-02 | |
| **GLUD1** | -0.119 | 4.07E-02 | Amino Acid Synthesis, Arginine Metabolism, Glutamine Metabolism, Mitochondrial Respiration |
| **NDUFS8** | 0.16 | 4.07E-02 | Mitochondrial Respiration, Reactive Oxygen Response |
| **GMPR2** | -0.144 | 4.10E-02 | Nucleotide Salvage, Nucleotide Synthesis |
| **GMPS** | -0.233 | 4.18E-02 | Nucleotide Synthesis |
| **EPC1** | 0.181 | 4.18E-02 | Epigenetic Regulation |
| **TK2** | 0.195 | 4.18E-02 | Nucleotide Salvage, Nucleotide Synthesis |
| **ITCH** | 0.113 | 4.45E-02 | Antigen Presentation, Endocytosis |
| **THBS1** | 0.392 | 4.52E-02 | Myc, PI3K |
| **ATP6V1F** | 0.149 | 4.53E-02 | Mitochondrial Respiration, mTOR |
| **FANCA** | -0.262 | 4.55E-02 | DNA Damage Repair |
| **SERINC5** | 0.202 | 4.63E-02 | Glutamine Metabolism |
| **RRAGC** | 0.198 | 4.66E-02 | Autophagy, mTOR, p53 Pathway |
| **GUSB** | -0.152 | 4.87E-02 | Lysosomal Degradation |

**MDA-MB-231 BI-99179 vs. DMSO**

| Gene | Log2 Fold Change | p* value | Gene Set |
|---|---|---|---|
| FASN | 0.852 | 1.41E-03 | AMPK, Fatty Acid Synthesis, Myc, Vitamin & Cofactor Metabolism |
| SLC25A1 | 0.43 | 2.77E-03 | Fatty Acid Synthesis |
| NCOA2 | 0.352 | 2.77E-03 | Epigenetic Regulation, Mitochondrial Respiration |
| GLUL | 0.245 | 2.77E-03 | Amino Acid Synthesis, Arginine Metabolism, Glutamine Metabolism |
| ACAT2 | 0.538 | 2.77E-03 | Amino Acid Synthesis, Fatty Acid Oxidation, TryptophanKynurenine Metabolism |
| PDP1 | -0.193 | 4.58E-03 | Mitochondrial Respiration |
| PDK4 | -0.997 | 4.58E-03 | Mitochondrial Respiration |
| GLUD1 | 0.175 | 4.58E-03 | Amino Acid Synthesis, Arginine Metabolism, Glutamine Metabolism, Mitochondrial Respiration |
| STAT3 | 0.22 | 4.80E-03 | Cytokine & Chemokine Signaling, Transcriptional Regulation |
| CPT1A | -0.732 | 1.35E-02 | AMPK, Fatty Acid Oxidation |
| GBA | 0.275 | 1.35E-02 | Lysosomal Degradation |
| PTGS2 | -0.469 | 1.35E-02 | Cytokine & Chemokine Signaling, NF-kB, Reactive Oxygen Response, Vitamin & Cofactor Metabolism |
| IDH1 | 0.288 | 1.35E-02 | IDH12 Activity, Reactive Oxygen Response, Vitamin & Cofactor Metabolism |
| COX8A | 0.143 | 1.35E-02 | Mitochondrial Respiration, p53 Pathway |
| SREBF2 | 0.318 | 1.35E-02 | Transcriptional Regulation |
| CYP1B1 | 0.37 | 1.35E-02 | TryptophanKynurenine Metabolism |
| TALDO1 | 0.145 | 1.35E-02 | Cytokine & Chemokine Signaling, Pentose Phosphate Pathway |
| TECR | 0.22 | 1.35E-02 | Fatty Acid Synthesis |
| HIF1A | 0.197 | 2.08E-02 | Autophagy, Cytokine & Chemokine Signaling, Hypoxia, Transcriptional Regulation |
| CTSD | 0.213 | 2.74E-02 | Antigen Presentation, Autophagy, Lysosomal Degradation |
| NQO1 | 0.282 | 3.25E-02 | KEAP1NRF2 Pathway, Reactive Oxygen Response |
| ACOX1 | 0.227 | 3.25E-02 | Fatty Acid Oxidation |
| SCD | 0.666 | 3.27E-02 | AMPK, Fatty Acid Synthesis |

| | | | |
|---|---|---|---|
| **PIK3CD** | 0.25 | 3.97E-02 | AMPK, Autophagy, Cytokine & Chemokine Signaling, mTOR, PI3K, TCR & Costimulatory Signaling |
| **MAP2K3** | 0.192 | 4.10E-02 | Cytokine & Chemokine Signaling, MAPK, TLR Signaling |
| **INSR** | 0.254 | 4.10E-02 | AMPK, MAPK, mTOR, PI3K |
| **SREBF1** | 0.294 | 4.10E-02 | AMPK, Transcriptional Regulation |
| **GABARAP** | 0.137 | 4.50E-02 | Autophagy |
| **MAT2A** | -0.157 | 4.70E-02 | Amino Acid Synthesis |
| **HEXA** | 0.202 | 4.95E-02 | Lysosomal Degradation |

PDO-BC25 BI-99179 vs. DMSO

| Gene | Log2 Fold Change | p* value | Gene Set |
|---|---|---|---|
| **KMT2E** | -0.206 | 1.80E-02 | Amino Acid Synthesis, Epigenetic Regulation, Transcriptional Regulation |
| **THBS2** | -0.367 | 3.25E-02 | PI3K |
| **SLC7A11** | 0.252 | 3.25E-02 | Amino Acid Transporters, Reactive Oxygen Response |
| **SCD** | 0.175 | 3.42E-02 | AMPK, Fatty Acid Synthesis |

**PDO-BC25-2 BI-99179 vs. DMSO**

| Gene | Log2 Fold Change | p* value | Gene Set |
|---|---|---|---|
| **FNIP2** | 0.421 | 1.06E-02 | mTOR |
| **PRKAA1** | 0.231 | 1.06E-02 | AMPK, Autophagy, mTOR, p53 Pathway, PI3K |
| **GBA** | 0.299 | 1.06E-02 | Lysosomal Degradation |
| **PSAT1** | 0.323 | 1.06E-02 | Amino Acid Synthesis, Glutamine Metabolism |
| **HMOX1** | 0.341 | 2.89E-02 | Cytokine & Chemokine Signaling, KEAP1NRF2 Pathway, Reactive Oxygen Response |
| **GLRX** | 0.241 | 2.90E-02 | Glutamine Metabolism |
| **SLC7A5** | 0.345 | 4.39E-02 | Amino Acid Transporters, mTOR |

**Supplemental Table 4:** Variants and copy number alterations of the PDO-BC25 and PDO-BC25-2 tumor tissues and cell lines.

| **Sample** | **Variants** | **Copy Number Alterations** |
|---|---|---|
| BC25 Tumor | *TP53* p.Y220H, *PIK3CA* p.H1047R, and *RAD51* p.E77fs | Deep deletion (two-copy loss) of *MTAP*/*CDKN2A*/*CDKN2B* |
| BC25 Cell Line | *TP53* p.Y220H, *PIK3CA* p.H1047R, and *RAD51* p.E77fs | Deep deletion (two-copy loss) of *MTAP*/*CDKN2A*/*CDKN2B* |
| BC25-2 Tumor | *TP53* p.Y220H, *PIK3CA* p.H1047R, and *RAD51* p.E77fs | Deep deletion (two-copy loss) of *MTAP*/*CDKN2A*/*CDKN2B* |
| BC25-2 Cell Line | *TP53* p.Y220H, *PIK3CA* p.H1047R, and *RAD51* p.E77fs | Deep deletion (two-copy loss) of *MTAP*/*CDKN2A*/*CDKN2B* |
